# Insights Into Olive Fruit Surface Functions: A Comparison of Cuticular Composition, Water Permeability, and Surface Topography in Nine Cultivars During Maturation

**DOI:** 10.3389/fpls.2019.01484

**Published:** 2019-11-19

**Authors:** Clara Diarte, Po-Han Lai, Hua Huang, Agustí Romero, Tomás Casero, Ferran Gatius, Jordi Graell, Vicente Medina, Andrew East, Markus Riederer, Isabel Lara

**Affiliations:** ^1^Universitat de Lleida, Lleida, Spain; ^2^Postharvest Unit-XaRTA, AGROTÈCNIO, Lleida, Spain; ^3^Massey Agrifood Technology Partnership, Massey University, Palmerston North, New Zealand; ^4^Julius-von-Sachs Institut für Biowissenschaften, Universität Würzburg, Würzburg, Germany; ^5^Oliviculture, Oil Science and Nuts, IRTA-Mas de Bover, Constantí, Spain; ^6^Applied Plant Biotechnology, AGROTÈCNIO, Lleida, Spain

**Keywords:** *Olea europaea* L., cuticular wax, cutin, maturity stage, water permeance, skin surface topography

## Abstract

Olive (*Olea europaea* L.) growing has outstanding economic relevance in Spain, the main olive oil producer and exporter in the world. Fruit skin properties are very relevant for fruit and oil quality, water loss, and susceptibility to mechanical damage, rots, and infestations, but limited research focus has been placed on the cuticle of intact olive fruit. In this work, fruit samples from nine olive cultivars (“Arbequina,” “Argudell,” “Empeltre,” “Farga,” “Manzanilla,” “Marfil,” “Morrut,” “Picual,” and “Sevillenca”) were harvested from an experimental orchard at three different ripening stages (green, turning, and ripe), and cuticular membranes were enzymatically isolated from fruit skin. The total contents of cuticular wax and cutin significantly differed among cultivars both in absolute and in relative terms. The wax to cutin ratio generally decreased along fruit maturation, with the exception of “Marfil” and “Picual.” In contrast, increased water permeance values in ripe fruit were observed uniquely for “Argudell,” “Morrut,” and “Marfil” fruit. The toluidine blue test revealed surface discontinuities on green samples of “Argudell,” “Empeltre,” “Manzanilla,” “Marfil,” and “Sevillenca” fruit, but not on “Arbequina,” “Farga,” “Morrut,” or “Picual.” No apparent relationship was found between water permeability and total wax coverage or the results of the toluidine blue test. The composition of cuticular waxes and cutin monomers was analyzed in detail, and sections of fruit pericarp were stained in Sudan IV for microscopy observations. Skin surface topography was also studied by means of fringe projection, showing large differences in surface roughness among the cultivars, “Farga” and “Morrut” fruits displaying the most irregular surfaces. Cultivar-related differences in cuticle and surface features of fruit are presented and discussed.

*Except the vine, there is no plant which bears a fruit of as great importance as the olive*. Pliny the Elder (*attributed*)

## Introduction

The olive (*Olea europaea* L.) tree is considered one of the oldest crops to have been domesticated by humans (Besnard et al., 2018). Around 90% of the world production of olives is used for the production of olive oil, and the rest is employed for the manufacture of table olives^1^. More than half of the total world olive production is grown in countries in the Mediterranean basin, Spain being the main olive oil producer and exporter in the world.

In Mediterranean areas, crops often develop under adverse environmental conditions, including restricted water availability, high temperatures, or elevated UV irradiation levels, which are expected to exacerbate in a scenario of global climate change. The performance of a given genotype under such conditions, as well as its resistance against pests and diseases, will be partly dependent upon the properties of fruit surface, which will act as the interface between the plant and the surrounding environment. Being the outer layer of the epidermis, the cuticle represents the first barrier against abiotic and biotic stress factors.

Plant cuticles are hydrophobic layers covering the epidermis of aerial, non-lignified plant organs, including the intact fruit. The cuticle scaffold is composed of the polyester cutin, an insoluble polymer matrix mostly containing hydroxy-, carboxy-, and epoxy-C_16_ and C_18_ fatty acids (Lequeu et al., 2003; Franke et al., 2005; Domínguez et al., 2011; Camacho-Vázquez et al., 2019). Different types of cuticular waxes, both in amorphous and crystalline form, and a variable amount of phenolics are integrated within or accumulate onto the surface of the cutin matrix (Samuels et al., 2008; Yeast and Rose, 2013; Lara et al., 2014). The cuticle is considered to limit transpirational water loss to prevent the desiccation of the fruit, but it also confers or modulates relevant properties such as the susceptibility to mechanical damage, infestations, and rots (Kunst and Samuels, 2002; Lara et al., 2014; Martin and Rose, 2014; Serrano et al., 2014; Riederer et al., 2015; Wang et al., 2016).

In spite of these considerations, very few published studies have addressed the composition of cuticles of intact olive fruit. Most of them have focused uniquely on cuticular waxes (Bianchi et al., 1992; Guinda et al., 2010; Vichi et al., 2016), whereas only one published study has also reported on cutin composition in fruit of this species (Huang et al., 2017). Compositional differences have been detected according to cultivar and maturation stage. These differences may relate to the water-proofing and mechanical properties of the cuticle, and thus be relevant for fruit resistance to abiotic and biotic stress-inducing factors.

In this study, olive fruit from nine oil- and table-cultivars differing in important quality traits were selected for the analysis of chemical composition and water permeability at three different maturity stages. With the purpose of widening the study on fruit surface differences across the considered genotypes, skin topography was non-destructively assessed by means of fringe projections (East et al., 2016). The bulk of results should help a better comprehension of the factors determining olive adaptations to the surrounding environment.

## Materials and Methods

### Plant Material and Toluidine Blue Test

Fruit samples from nine olive (*Olea europaea* L.) autochthonous Spanish cultivars (“Arbequina,” “Argudell,” “Empeltre,” “Farga,” “Manzanilla,” “Marfil,” “Morrut,” “Picual,” and “Sevillenca”) were hand-collected at an experimental orchard located at IRTA-Mas Bové (Constantí, Spain; 41°09’ N, 1°12’ E; altitude 100 m) from trees supplied with drip irrigation. Annual rainfall is 500 mm, and takes place mainly in April–May and September. Fertilization and cultural practices at the orchard are the usual in the producing area. Olives were picked at three different maturity stages (green, turning, and ripe) based on skin color during the usual harvest period (September to December) in 2016. Maturity index (0–7), fresh weight (g), flesh-to-stone ratio, and water content (% humidity) were determined on 50 fruits per cultivar and maturity stage. For the assessment of length and diameter (mm), 10 fruit were used (**Table 1**). Maturity index was scored on a 0–7 scale by subjectively categorizing each fruit within the sample according to skin and flesh color (Uceda and Frías, 1975); values indicate the weighted average of the 50 olives examined. Olive fly infestation was likewise assessed on 50 fruits per cultivar and maturity stage, by visually checking each fruit for egg deposition, and data shown as a percentage (**Table S1**). In order to visualize possible discontinuities on fruit surface, samples of fresh olives at the green stage (10 fruits per cultivar) were stained in a toluidine blue (TB) solution (0.05%, w/v) for 2 h (Tanaka et al., 2004), rinsed, and allowed to dry in air. Since ripe “Marfil” fruit turn white rather than black, the TB test was applied also to these samples.

**Table 1:** Physical characteristics and toluidine blue test of olive fruits used in this study.

Cultivar	Maturity stage	Sampling date	Maturity Index	Weight (g)	F:S ratio^*^	Water content (%)	Length (mm)		Diameter (mm)		TB test*
**“Arbequina”**	Green	Sept 29	0.26	1.10	2.68	53.9	14.1	ab D	12.1	b CD	−
	Turning	Sept 29	2.14	1.27	3.18	55.2	13.4	b C	12.0	b B	ne
	Ripe	Nov 27	3.40	1.59	4.24	58.2	14.7	a D	13.0	a DE	ne
**“Argudell”**	Green	Sept 29	0.26	2.02	4.08	56.0	18.6	a C	13.9	b BC	+
	Turning	Nov 27	0.96	2.65	5.32	59.2	20.1	a B	15.3	ab A	ne
	Ripe	Nov 27	2.36	2.81	5.57	59.6	20.0	a BC	15.9	a B	ne
**“Empeltre”**	Green	Sept 29	0.48	3.18	4.05	56.1	23.7	a A	15.2	a B	+
	Turning	Sept 29	3.58	3.09	4.40	55.4	23.0	a A	15.0	a A	ne
	Ripe	Nov 27	5.00	3.13	4.00	49.3	24.1	a A	15.0	a BCD	ne
**“Farga”**	Green	Sept 29	0.36	1.28	2.47	54.8	16.9	b CD	10.8	b C	−
	Turning	Sept 29	2.04	1.74	3.18	58.7	19.0	a B	12.5	a B	ne
	Ripe	Nov 27	4.40	1.82	3.70	55.8	18.1	a C	12.0	a E	ne
**“Manzanilla”**	Green	Sept 29	0.12	4.57	8.31	70.1	24.0	a A	18.6	a A	+
	Ripe	Nov 27	5.88	4.65	7.68	66.6	23.9	a A	19.4	a A	ne
**“Marfil”**	Green	Sept 29	0.04	1.32	2.05	60.1	19.7	b BC	10.5	b C	+
	Ripe	Dec 12	0.96	1.98	3.95	53.6	21.6	a AB	13.3	a CDE	+
**“Morrut”**	Green	Sept 29	0.16	1.99	2.03	51.6	20.4	b BC	13.8	b BC	−
	Turning	Nov 27	1.04	2.34	2.52	51.1	20.4	b B	13.8	b A	ne
	Ripe	Jan 16	3.40	2.08	2.74	37.6	21.8	a AB	15.5	a BC	ne
**“Picual”**	Green	Sept 29	0.30	2.72	2.75	57.2	22.4	a AB	15.1	a B	−
	Turning	Nov 27	2.84	3.06	3.11	60.2	22.2	a AB	15.3	a A	ne
	Ripe	Nov 27	3.88	4.30	4.35	49.6	24.1	a A	17.3	a AB	ne
**“Sevillenca”**	Green	Sept 29	0.32	2.71	3.09	56.7	21.4	a ABC	14.1	a BC	+
	Ripe	Nov 27	3.16	3.32	4.97	52.0	22.1	a AB	15.5	a BC	ne

Values represent means of 50 fruits for maturity index, weight, F:S ratio, and water content, and of 10 fruits for length, diameter, and TB test. Different capital letters denote significant differences among the cultivars for a given maturation stage, and different lower-case letters stand for significant differences among maturation stages for a given cultivar, at P ≤ 0.05 (Student’s t-test). Fruit weight, F:S ratio, and water content were determined jointly for 50 fruits, and values reported represent the average of the 50 olives assessed. Maturity index values indicate the weighted average of the 50 olives within the sample (Uceda and Frías, 1975).

*F:S ratio, flesh to stone ratio; TB test, toluidine blue test (Tanaka et al., 2004): stained and non-stained fruits are denoted respectively as + and −; ne, not evaluated.

### Cuticle Isolation

Disks of fruit exocarp (two disks per fruit) were excised with a cork borer. Thirty to 75 olive s, depending on fruit size, were processed so to obtain around 100 cm^2^ of skin per cultivar and maturity stage as described elsewhere (Belge et al., 2014a). Because not enough skin sample can be obtained from one individual olive fruit to enable further analysis of cuticle composition, excised skin disks were pooled into one sample of biological material. Exocarp samples were distributed in two tubes (50 cm^2^ per tube) for the enzymatic isolation of cuticular membranes (CM). Disks were incubated at 37°C in cellulase/pectinase solution (0.2% (w/v) cellulase, 100 U ml^−1^ pectinase, and 1 mM NaN_3_ in 50 mM citrate buffer at pH 4.0 until no more material was released, and then washed in citrate buffer (50 mM, pH 4.0) until no material was left in suspension. After thoroughly rinsing in distilled water, CM disks were dried at 40°C, weighted, and then pooled and kept in hermetically capped vials until analysis. Cuticle yields were expressed per unit of fruit surface area (µg cm^−2^).

### Extraction and Analysis of Cuticular Wax

CM samples (20 mg/replicate × 3 technical replicates) were dewaxed in chloroform (2 mg ml^−1^) for 24 h at room temperature, with constant shaking. Chloroform extraction was done three times, and the chloroform extracts were pooled, incubated 15 min in an ultrasonic bath and filtered. Dewaxed CM (DCM) were dried and kept in hermetically capped vials for subsequent analysis of cutin monomers. The chloroform extracts were concentrated at 40°C using a rotatory evaporator, and waxes then transferred to a pre-weighed vial, dried in a vacuum concentrator at 40°C until complete dryness, and weighed to calculate total wax yields (µg cm^−2^). Dotriacontane (C_32_) was then added as an internal standard, and samples were derivatized during 15 min at 100°C in *N,O*-bis(trimethylsilyl)trifluoroacetamide (BSTFA) and pyridine (3:2, v/v), in order to obtain trimethylsilyl (TMSi) ethers and esters from free hydroxyl and carboxyl groups, respectively.

Wax samples (1 µl) were injected in on-column mode into a gas chromatography-mass spectrometry (GC-MS) system for compound identification and quantification. This GC equipment (Agilent 7890N) was coupled with a quadrupole mass selective detector (Agilent 5973N) and equipped with a capillary column (DB 5 MS UI, 30 m × 0.25 mm × 0.25 µm; SGE Europe Ltd., Milton Keynes, UK). Compounds were identified by comparison with their retention times with those of standards, and through their electron ionization-mass spectra using a mass spectral library (NIST 11 MS). Chromatographic conditions were as follows: oven was set at 100°C for 1 min, then raised by 15°C min^−1^ to 200°C, then by 5°C min^−1^ to 310°C, and finally held 10 min at 310°C. Helium was used as the carrier gas at 1.0 ml min^−1^. A flame ionization detector (FID) was used for quantitative analysis of cuticular waxes, in the same chromatographic conditions as described above excepting that, at the last step, the oven was held at 310°C for 13 min and that a higher carrier gas flow (1.3 ml min^−1^) was used. Data are expressed as a relative percentage (% over total waxes). Average chain length (ACL) of acyclic wax compounds was calculated as the weighted average number of carbon atoms, defined as

$$ACL=\frac{\Sigma C_{n}n}{\Sigma C_{n}}$$

where *C_n_* is the percentage of each acyclic wax compound with *n* carbon atoms.

### Extraction and Analysis of Cutin Monomers

DCM samples (roughly 10 mg/replicate × 3 technical replicates) were hydrolyzed for 2 h in 2 ml of 1 M HCl in 100% MeOH, esterified in the same solution during 2 h at 80°C, and added 2 ml saturated NaCl after cooling down. Cutin monomers were extracted three consecutive times in 2 ml hexane for 10 min using a mixer and centrifuged at 20°C. The collected supernatants were pooled and transferred into a pre-weighed vial, dried completely using a vacuum concentrator at 40°C, and then weighed to calculate total cutin yields (µg cm^−2^). Heptadecanoate (C_17_) and tricosanoate (C_23_) were added as internal standards, and then samples were derivatized during 15 min at 100°C in BSTFA and pyridine (3:2, v/v). Derivatized samples (1 µl) were finally injected in on-column mode into a GC-MS and a GC-FID system for compound identification and quantification, respectively, under the same chromatographic conditions as described above for the analysis of cuticular waxes.

### Determination of Cuticular Transpiration

Transpiration from the whole fruit was determined gravimetrically from measures of water loss over time as described elsewhere (Huang et al., 2017). Eight to twelve olives per sample were sealed with paraffin wax on the pedicel area (melting point 65°C; Roth, Karlsruhe, Germany). To reduce the relative humidity until approximately zero, fruit samples were placed in boxes over silica gel (AppliChem, Darmstadt, Germany) and kept at 25°C in an incubator (IPP 110, Memmert, Schwabach, Germany). Weight loss of the samples was monitored over time (five to six data points per individual sample) with an analytic electronic balance with ± 0.1 mg precision (MC-1 AC210S, Sartorius, Göttingen, Germany). Temperature inside the incubator was controlled continuously with a digital thermometer (Testoterm 6010, Lenzkirch, Germany) and the actual fruit temperature was measured using an infrared laser thermometer (Harbor Freight Tools, Calabasas, California). Transpiration rates (flux of water vapor; *J* in g m^−2^ s^−1^) of the samples were calculated from changes in the fresh weight (Δ*W* in g) over time (Δ*t* in s) and surface area (A in m^2^) as indicated below:

$$J=\frac{∆W}{∆t\cdot A}$$

The permeance (*P* in m s^−1^) was calculated from the transpiration rate (*J*) divided by the driving force:

$$P=\frac{J}{c_{ww}^{*}(a_{fruit}-a_{air})}$$

where c*_wv_ was the water vapor content of air at saturation, obtained from tabulated values, *a_fruit_* was the water activity in the fruit, which was assumed to be unity, and *a_air_* was air water activity (that was close to zero).

### Skin Surface Topography

Micro-topography of samples (25 olives per cultivar) was captured at two locations (180° apart) on the equatorial area of each individual fruit using fringe projection equipment (Primos™ Lite, Cranfield Image System, USA). Topography data were collected with an x-y resolution of 26.83 µm and z (vertical) resolution of 2 µm. Subsequent calculations to extract surface roughness descriptive parameters (Lai et al., 2018) were conducted with the accompanying proprietary software package (Primos™ v5.8, Cranfield Image Systems, USA).

Surface roughness parameters studied in this work were *Sa*, *Stm*, *Spm*, *Svm*, *Sk*, and *S* (Gadelmawla et al., 2002). *Sa* is the arithmetic average height parameter, defined as the mean of the absolute deviation of roughness irregularities from the mean line. *Stm* describes the mean distance between the lowest valley and the highest peak at the measured area. *Spm* is defined as the mean of the maximum height peaks, and *Svm* is the mean of the maximum depth valleys. *Sk* measures peak-to-valley surface roughness after excluding the predominant peaks and valleys, and hence illustrates the core roughness depth. *S* is the only horizontal parameter, defined as the average spacing between profile peaks at the mean line in the profile measured.

### Microscopy Observations

Pericarp fruit samples were chopped to little cubes (roughly 2 mm per side) and fixed in a formaldehyde-acetic acid (FAA) solution [5% (v/v) formaldehyde and 5% (v/v) glacial acetic acid in 1:1 (v/v) ethanol-distilled water] for 12 h. Samples were dehydrated in aqueous solutions containing increasing ethanol concentrations up to 100% (v/v). Dehydrated samples were transferred to Eppendorf tubes for infiltration and polymerization in Technovit 7100^®^ resin (Heraeus Kulzer GmbH, Wehrheim, Germany), and the resin was dried at 45°C for 24 h.

Resin-embedded samples were cut in 4-µm-thick sections using an ultramicrotome (Leica EM UC6, Leica Microsystems GmbH, Wetzlar, Germany), and subsequently stained on a slide for 15 min in a Sudan IV lysochrome solution [5% (w/v) in 85% (v/v) ethanol] in order to visualize the lipidic constituents of fruit cuticles. Excess staining was removed by rinsing in 50% (v/v) ethanol, and samples allowed to dry at room temperature. Olive pericarp sections were observed and photographed using a microscope (Leica DM4000 B) with a coupled camera (Leica DFC300 FX). Cuticle thickness was determined from five images obtained from five different fruit per cultivar and maturity stage with the Fiji image processing software (Schindelin et al., 2012).

### Statistical Analysis

The statistical analyses were conducted with the JMP^®^ Pro 13 software. Results were calculated as means ± standard deviations. Multifactorial analysis of variance (ANOVA) procedures were applied, with cultivar and maturation stage as the factors, and means were compared with the Student’s *t* test (*p* ≤ 0.05). PCA was used to help the interpretation of the data set obtained, using the Unscrambler software, version 9.1.2 (CAMO ASA, Oslo, Norway). Data were centered and weighed by the inverse of the standard deviation of each variable, and full cross-validation was run as a validation procedure.

## Results

Olive cultivars assessed in this study included some preferentially used for oil production (“Argudell,” “Picual,” “Sevillenca”), for manufacturing of table olives (“Empeltre,” “Manzanilla”), or for both purposes. The choice of genotypes comprised representatives of very early (“Empeltre,” “Manzanilla”), early (“Sevillenca”), medium (“Arbequina,” “Argudell,” “Farga,” “Picual”), and late (“Marfil,” “Morrut”) ripening patterns (Tous and Romero, 1993), as well as a range of fruit sizes (**Table 1**). Due to differing ripening patterns, data corresponding to the turning stage are lacking for three cultivars (“Manzanilla,” “Marfil,” and “Sevillenca”), as not enough fruit material was found at the sampling dates. The highest flesh to stone ratios, weight, and water contents were found for “Manzanilla,” a very common table olive cultivar in Spain. The highest incidence of olive fly infestation was observed for “Empeltre” and “Manzanilla,” which showed very high percentages of affected fruits, particularly in unripe olives (**Table S1**).

### Surface Differences

Differences in surface characteristics were found among the nine olive cultivars assessed. Green fruits were stained with toluidine blue in order to visualize pores, cracks, or defects on the surface. Two groups of cultivars were revealed by the TB test: fruits of “Argudell,” “Empeltre,” “Manzanilla,” “Marfil,” and “Sevillenca” were stained, whereas those of “Arbequina,” “Farga,” “Morrut,” and “Picual” were not, even after leaving the fruit samples in the staining solution for several hours.

**Figure 1:**
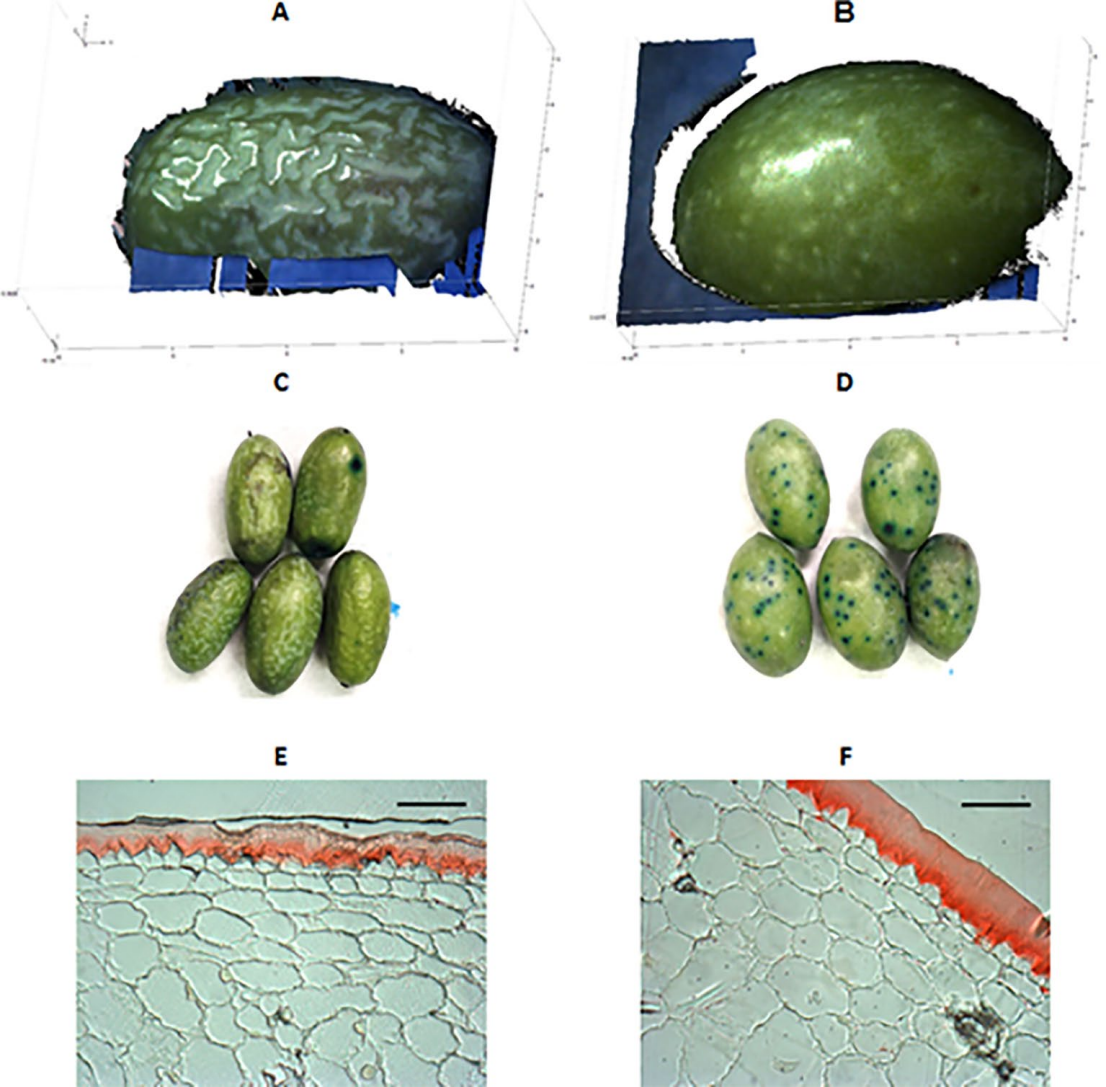
An example of fruit surface differences between two olive cultivars (left, “Farga”; right, “Sevillenca”) at the green stage. **(A**, **B)**: 3D-diagrams of raw data outputs from fringe projections. Blue and black areas represent background noise due to the shape and size of the olives, which did not cover the whole assessment window of the equipment. **(C**, **D)**: Toluidine blue (TB) staining. **(E**, **F)**: Sudan IV-stained cross-sections of fruit pericarp observed under a bright-field microscope (bar: 60 μm).

Differences in skin topography of green fruit among the cultivars were also detected. “Farga” and “Morrut” fruits showed the most irregular surface as shown by higher values of vertical roughness parameters (*Sa*, *Stm*, *Spm*, *Svm*, and *Sk*). On the contrary, fruit of “Sevillenca,” ‘Empeltre,” “Arbequina,” and “Argudell” had smoother skin surface than other cultivars based on lower values of vertical parameters together with higher horizontal roughness as shown by *S*, representative of peak-to-peak spacing (**Table 2**). “Farga” samples displayed very different micro-topography and visual appearance as revealed by fringe projection data in comparison with other cultivars. In order to highlight the distinctive features of the surface of “Farga” olives, a boxplot of *Sa* as the most common roughness parameter is provided (**Figure S1**). Three-dimensional diagrams of raw data obtained from fringe projections, TB staining, and micrographs of Sudan IV-stained pericarp cross-sections for “Farga” and “Sevillenca” fruit are shown as an example to illustrate these differences (**Figure 1**), while results for the rest of cultivars assessed are presented as supplementary figures (**Figures S2, S3**, and **S4** respectively). The surface of “Sevillenca” green olives was not only smoother than that of “Farga” at the same maturity stage, but also displayed significantly thicker cuticles (**Table 3** and **Figure 1**).

**Table 2:** Surface roughness parameters (μm) measured in olive fruits at the green stage.

Cultivar	*Sa*	*Stm*	*Spm*	*Svm*	*Sk*	*S*
**“Arbequina”**	8.5 de	54.4 d	25.4 d	−29.0 a	27.0 de	839.4 a
**“Argudell”**	8.5 de	58.4 d	27.8 d	−30.6 a	27.0 de	772.6 bc
**“Empeltre”**	7.4 e	51.3 d	24.3 d	−27.1 a	23.5 e	818.2 ab
**“Farga”**	20.6 a	98.7 a	49.8 a	−48.9 c	60.2 a	678.6 e
**“Manzanilla”**	11.2 bc	76.5 bc	36.8 bc	−39.7 b	36.8 b	699.0 de
**“Marfil”**	9.8 cd	69.1 c	33.4 c	−35.7 b	30.5 cd	735.1 cd
**“Morrut”**	12.8 b	80.7 b	40.7 b	−40.0 b	39.3 b	654.8 e
**“Picual”**	10.0 cd	72.7 bc	34.8 c	−37.9 b	32.1 c	678.5 e
**“Sevillenca”**	7.4 e	51.4 d	24.7 d	−26.7 a	23.5 e	739.5 cd

Sa, Stm, Spm, and Svm are related to vertical roughness, Sk represents core roughness, and S stands for horizontal roughness. Values represent means of 25 olives per cultivar. Means followed by different letters within a column are significantly different at P ≤ 0.05 (Student’s t test).

### Cuticle Characteristics and Changes Along Maturation

With the exception of “Arbequina,” cuticle yields (mg cm^−2^ surface area) did not change significantly along fruit maturation (**Table 3**). Total cuticle amounts at the green and the ripe stages ranged from 1.9 to 3.1 mg cm^−2^ and from 2.0 to 3.8 mg cm^−2^, respectively. At both maturity stages, the lowest yields were observed for “Manzanilla” fruit. Consistent with the lowest cuticle yields, “Manzanilla” olives also displayed the lowest values for cuticle thickness, irrespective of maturity stage. Cuticle thickness remained steady along on-tree maturation in five (“Argudell,” “Farga,” “Manzanilla,” “Marfil,” and “Picual”) out of the nine cultivars studied, while a significant decrease was observed for the rest of the genotypes (“Arbequina,” “Empeltre,” “Morrut,” and “Sevillenca”) (**Table 3**).

**Table 3:** Total cuticle amounts, cuticular wax and cutin yields, wax to cutin ratios, cuticle thickness, and water permeance in olive fruits at the green, turning, and ripe stages.

Cultivar	Maturity stage	Cuticle yield (mg cm^−2^)		Wax yield (μg cm^−2^)		Wax (%)		Cutin yield (μg cm^−2^)		Cutin (%)		C16/C18	Wax/cutin ratio		Thickness (μm)		Permeance (× 10^−5^ m s^−1^)
**“Arbequina”**	Green	3.1	a A	1163.6	a A	37.4	a AB	1205.4	a A	38.7	a A	0.41	0.97	a B	41.0	ab B	7.3
	Turning	2.4	b DE	405.9	c D	16.7	c C	945.0	b BC	39.0	a A	0.33	0.43	c C	44.5	a A	7.9
	Ripe	2.5	ab B	456.6	b D	18.5	b D	931.1	b BC	37.7	a AB	0.37	0.49	b EF	32.9	b BC	8.0
**“Argudell”**	Green	2.9	a AB	649.4	a D	22.8	a E	846.9	b BC	29.8	b D	0.43	0.77	a C	35.0	a BC	11.2
	Turning	3.6	a A	497.4	b C	13.7	c D	995.5	a B	27.4	b B	0.41	0.50	b C	34.2	a ABC	10.8
	Ripe	2.7	a AB	453.5	b D	16.7	b DE	1021.4	a B	37.7	a AB	0.40	0.45	b F	36.3	a AB	11.9
**“Empeltre”**	Green	2.3	a BCDE	903.6	a C	39.1	a A	722.7	b DE	31.3	a CD	0.35	1.26	a A	62.9	a A	na
	Turning	2.8	a CD	882.6	a A	31.6	b A	826.0	ab CD	29.6	a B	0.44	1.08	a A	38.7	b ABC	na
	Ripe	3.0	a AB	496.1	b CD	16.6	c DE	890.5	a BCD	29.9	a CD	0.38	0.56	b DE	32.9	b BC	7.4
**“Farga”**	Green	2.7	a ABCD	899.3	a C	34.0	a C	962.3	a B	36.4	a ABC	0.49	0.94	a BC	26.2	a C	na
	Turning	2.3	a E	564.6	b BC	24.9	b B	807.8	b CD	35.7	a A	0.44	0.70	b B	33.6	a BC	na
	Ripe	2.4	a B	229.7	c F	9.8	c F	789.1	b CD	33.5	a BC	0.37	0.29	c G	27.7	a BC	7.3
**“Manzanilla”**	Green	1.9	a E	566.3	a E	30.2	a D	620.1	a E	33.1	a BCD	0.44	0.92	a BC	25.5	a C	na
	Turning	na		na		na		na		na		na	na		28.5	a C	na
	Ripe	2.0	a B	364.7	b E	18.1	b D	527.5	a E	26.2	a D	0.43	0.69	b BC	25.7	a C	7.2
**“Marfil”**	Green	2.1	a CDE	320.2	b F	14.7	b F	810.0	a CD	37.3	a AB	0.29	0.40	b D	34.4	a BC	na
	Turning	na		na		na		na		na		na	na		32.8	a C	na
	Ripe	2.3	a B	581.0	a B	25.4	a B	756.1	a D	33.0	a BC	0.28	0.78	a AB	31.0	a BC	11.8
**“Morrut”**	Green	3.0	a A	994.8	a B	33.6	a C	933.9	a B	31.6	a CD	0.27	1.07	a B	44.4	a B	6.9
	Turning	3.0	a BC	371.8	c D	12.2	c D	781.4	a D	25.7	b B	0.23	0.48	b C	32.2	b C	9.4
	Ripe	2.8	a AB	437.5	b D	15.5	b E	886.1	a BCD	31.5	a CD	0.32	0.49	b EF	35.8	ab AB	11.1
**“Picual”**	Green	2.7	a ABC	973.1	b BC	35.8	a BC	952.2	c B	35.1	a ABC	0.36	1.03	a B	37.0	a B	6.6
	Turning	3.4	a AB	635.7	c B	19.0	b C	1230.9	b A	36.8	a A	0.32	0.52	c C	43.9	a AB	7.3
	Ripe	3.8	a A	1284.5	a A	33.5	a A	1543.9	a A	40.2	a A	0.33	0.83	b A	44.3	a A	8.1
**“Sevillenca”**	Green	2.1	a DE	693.0	a D	33.2	a C	668.6	a E	31.9	a CD	0.41	1.05	a B	40.6	a B	7.3
	Turning	na		na		na		na		na		na	na		31.6	b BC	7.6
	Ripe	2.4	a B	535.8	b BC	21.9	b C	834.8	a CD	34.2	a BC	0.36	0.65	b CD	30.8	b BC	7.3

Cuticular membranes were isolated from skin samples (around 100 cm^2^) obtained from 30 to 75 olives, contingent upon fruit size. Wax and cutin data represent means of three technical replicates of this starting material. For cuticle thickness and water permeance, values represent means of five or 10 biological replicates, respectively (na, value not available). Different capital letters denote significant differences among the cultivars for a given maturity stage, and different lower-case letters stand for significant differences among maturity stages for a given cultivar, at P ≤ 0.05 (Student’s t test).

* Ratio of C_16_ to C_18_ cutin monomers.

Water permeance was determined in mature fruit of all nine cultivars studied. Two cultivar types could be defined according to permeability levels: a low-permeance group, including “Arbequina,” “Empeltre,” “Farga,” “Manzanilla,” “Picual,” and “Sevillenca” and displaying water permeance values ranging from 7.21 (“Manzanilla”) to 8.13 (“Picual”) × 10^−5^ m s^−1^, and a high-permeance cultivar set (“Argudell,” “Marfil,” and “Morrut”) showing water permeance values above 11 × 10^−5^ m s^−1^ (**Table 3**). No changes in water permeance levels were observed along maturation for “Arbequina,” “Argudell,” or “Sevillenca,” while significant increases in mature as compared to green fruit were found for “Morrut” and “Picual” (38 and 19%, respectively).

Excepting “Marfil” and “Picual,” wax yields decreased along maturation, both in absolute terms (µg cm^−2^) and as a percentage over total cuticle (**Table 3**). Wax percentages ranged from 9.8% in “Farga” mature fruit to 39.1% in “Empeltre” green samples. When expressed as mass per surface area, yields ranged from roughly 230 µg cm^−2^ in “Farga” mature olives to over fivefold that much (1,284.5 µg cm^−2^) in “Picual” mature fruit, consistent with thicker cuticles in these samples (**Table 3**). More cultivar-to-cultivar variation was observed for cutin, both regarding yields and time-course changes along on-tree maturation. Total cutin yields decreased over fruit maturation in “Arbequina” and “Farga” fruits (approximately 23 and 18%, respectively), whereas they increased in “Picual,” “Argudell,” and “Empeltre” fruits (by 38, 19, and 17%, in that order) and remained steady in the rest of the considered cultivars (“Manzanilla,” “Marfil,” “Morrut,” and “Sevillenca”). Wax-to-cutin ratio declined with maturity stage in all cultivars with the exception of “Marfil,” owing to increased wax contents.

### Cuticular Wax Composition

Triterpenoids were the dominant fraction in cuticular waxes, relative percentages over total waxes ranging from 58 to roughly 81% (**Table 4**). For “Farga,” “Marfil,” and “Picual” olives, the total amount of triterpenoids decreased with maturity by 15, 18, and 23%, respectively, whereas no significant changes were observed for the rest of the cultivars considered. Maslinic (27 to 52%, contingent on cultivar and maturity stage) and oleanolic (19 to 43%) acids were detected in the wax fraction obtained from all the samples, with very minor contents of ursolic acid being identified additionally in “Farga,” “Picual,” and “Sevillenca” fruit (**Supplementary Table 2**). Relative contents of oleanolic acid decreased with maturity stage, while the amounts of maslinic acid generally showed limited changes, with the exception of “Farga” and “Picual” olives which displayed a sustained decline over maturation, and “Sevillenca” samples for which, on the contrary, increased maslinic acid contents were found for the ripe as compared with the green stages.

**Table 4:** Relative amounts (% over total waxes) of wax compound types in cuticles isolated from olive fruits at the green, turning, and ripe stages.

Cultivar	Maturity stage	ACL *		Acyclic/cyclic ratio		Triterpenoids (%)		Fatty acids (%)		Fatty alcohols (%)		*n*-Alkanes (%)		Sterols (%)		Unidentified (%)	
**“Arbequina”**	Green	25.2	a AB	0.30	a BC	63.7	a CDE	7.4	a B	8.0	a BC	3.7	a A	1.1	a A	16.2	a AB
	Turning	25.0	a A	0.25	a ABC	68.3	a AB	9.0	a C	4.7	a AB	3.1	ab A	0.4	a B	14.5	a B
	Ripe	24.6	a BC	0.26	a CDE	67.6	a AB	9.6	a C	5.7	a C	2.3	b CD	0.8	a BCD	14.0	a BC
**“Argudell”**	Green	25.2	a AB	0.18	a D	70.0	a BCD	4.4	b CD	5.2	a D	3.2	a AB	0.9	a AB	16.4	a AB
	Turning	23.7	b B	0.21	a BC	64.7	a B	8.4	a CD	2.8	b B	2.6	a AB	1.1	a AB	20.5	a A
	Ripe	24.0	ab E	0.22	a DE	66.2	a AB	8.4	a C	3.4	b E	3.2	a BC	1.7	a A	17.1	a BC
**“Empeltre”**	Green	24.9	a AB	0.17	a D	71.3	a BC	5.2	b BCD	5.1	ab D	2.2	a BC	0.9	ab AB	15.4	a BC
	Turning	24.8	a A	0.19	a BC	73.3	a A	5.8	b E	7.0	a A	0.6	b D	0.4	b B	12.8	a B
	Ripe	24.2	b DE	0.22	a DE	70.2	a A	9.3	a C	3.6	b DE	2.7	a BCD	1.2	a ABC	13.0	a BC
**“Farga”**	Green	25.6	a A	0.20	b CD	73.5	a AB	6.9	b BC	6.0	a CD	2.0	ab CD	0.5	ab BC	11.1	b CD
	Turning	24.9	ab A	0.26	ab AB	70.6	a AB	11.0	a B	6.3	a A	1.3	b CD	0.4	b B	10.4	b B
	Ripe	24.4	b CD	0.33	a BC	62.9	b AB	12.7	a B	4.6	a CD	3.5	a B	0.8	a BCD	15.5	a BC
**“Manzanilla”**	Green	25.0	a AB	0.41	a AB	63.1	a DE	13.0	a A	11.5	a A	0.5	b E	0.38	a CD	11.6	a CD
	Ripe	24.9	b B	0.49	a A	58.4	a B	15. 8	a A	10.7	a A	2.0	a D	0.8	a BCD	12.4	a CD
**“Marfil”**	Green	25.5	a A	0.19	b D	74.8	a AB	7.3	b B	5.8	b CD	1.0	b DE	0.6	a BC	10.6	a D
	Ripe	25.4	a A	0.40	a AB	61.6	b AB	11.9	a B	8.1	a B	4.8	a A	0.6	a CD	13.1	a BC
**“Morrut”**	Green	23.9	b C	0.13	b D	70.7	a BCD	6.2	b BC	2.5	c E	0.5	b E	0.2	b CD	20.0	a A
	Turning	23.9	b B	0.31	a A	64.1	a B	15.2	a A	3.3	b B	2.0	a BC	1.5	a A	14.0	b B
	Ripe	24.7	a B	0.30	a CD	67.2	a AB	12.7	a B	5.0	a C	2.2	a CD	0.4	ab D	12.5	b C
**“Picual”**	Green	24.6	a BC	0.09	b D	80.8	a A	2.8	c D	4.5	a DE	0.2	b E	nd	b D	11.7	b CD
	Turning	23.9	b B	0.17	a C	72.1	ab AB	6.7	a DE	3.3	a BC	2.2	a B	1.5	a A	14.2	b B
	Ripe	24.2	b DE	0.16	a E	61.9	b AB	4.6	b D	3.6	a DE	1.8	a D	1.2	a ABC	26.9	a A
**“Sevillenca”**	Green	25.5	a A	0.44	a A	61.5	a E	15.7	a A	9.9	a AB	1.7	a CD	0.9	a AB	10.4	b D
	Ripe	24.4	b CD	0.34	a BC	58.1	a B	15.1	a A	3.0	b E	2.3	a CD	1.5	a AB	20.1	a AB

Cuticular membranes were isolated from skin samples (around 100 cm^2^) obtained from 30 to 75 olives, contingent upon fruit size. Values represent means of three technical replicates of this starting material (nd, non-detectable). Different capital letters denote significant differences among the cultivars for a given maturity stage, and different lower-case letters stand for significant differences among maturity stages for a given cultivar, at P ≤ 0.05 (Student’s t test).

^*^ ACL, average chain length of acyclic wax compounds.

Reduction of triterpenoid contents along maturation in “Farga,” “Marfil,” and “Picual” fruits, together with an increment in fatty acids, led to increased acyclic to cyclic compounds ratios (**Table 4**). Augmented percentages of fatty acids, alcohols, and *n*-alkanes over total waxes during maturation also caused increased acyclic to cyclic compounds ratios in “Morrut” samples, whereas the increase in fatty acids observed in “Argudell” and “Empeltre” was not important enough to significantly modify this ratio. Fatty acids and alcohols were the main types of acyclic compounds identified in cuticular waxes, with very minor percentages of *n*-alkanes, in contrast with reports for other fruit species, for which much higher *n*-alkane percentages in cuticular waxes have been reported (Belge et al., 2014a; Belge et al., 2014b). “Manzanilla” and “Sevillenca” samples displayed the highest relative percentages of fatty acids and alcohols (**Table 4**). Among fatty acids, the most abundant compounds detected lignoceric (24:0) and cerotic (26:0) acids, while tetracosanol (C_24_) and hexacosanol (C_26_) were the predominant alcohols (**Table S2**). The ACL of the acyclic compounds identified in the wax fraction decreased in the course of fruit maturation for most of the cultivars included in this work, with the exception of “Arbequina” and “Marfil,” for which no significant differences were found, and “Morrut” which showed an increase from the turning to the ripe stages (**Table 4**).

### Cutin Monomer Composition

C_18_-type monomers stood out quantitatively in cutin composition of the cultivars considered, representing around two thirds (68.7 to 76.2%) over total cutin monomers identified (**Supplementary Table 3**). The predominant compound type detected in the cutin fraction was hydroxy-fatty acids, relative percentages ranging 40 to 59% (**Table 5**). Among these, *ω*-hydroxy fatty acids and *ω*-hydroxy fatty acids with midchain hydroxyl groups were particularly abundant, mainly 18-hydroxyoctadecenoic and 16-hydroxyhexadecanoic acids. The relative percentage of 18-hydroxyoctadecenoic showed in general a moderate decline along maturation, “Picual” samples displaying the highest contents of this cutin monomer (24.1, 21.5, and 22.5% at the green, turning, and black stage, respectively). A similar trend, with the exception of ‘Morrut,” was observed for 16-hydroxyhexadecanoic acid, “Manzanilla” and “Picual” fruits showing the highest amounts of this compound. The relative percentages of ω-hydroxy fatty acids with midchain hydroxyl groups remained steady throughout fruit maturation, “Farga” samples showing the highest values (29.4% at the green stage) (**Table 5**). The predominant cutin monomer of this type was 9/10,16-dihydroxyhexadecanoic acid, which did not show noticeable variations during fruit maturation (**Table S3**).

**Table 5:** Relative amounts (% over total cutin) of cutin monomer types in cuticles isolated from olive fruits at the green, turning, and ripe stages.

**Cultivar**	**Maturity stage**	FA* **(%)**		**α,ω-diFA, mcOH (%)**		**α,ω-diFA, mcOH (%)**		**ω-OH FA (%)**		**ω-OH FA, mcOH (%)**		**α-OH FA (%)**		**Other OH FA** **(%)**		**Alcohols** **(%)**		**Unidentified** **(%)**	
**“Arbequina”**	Green	5.0	b BC	11.5	a D	2.6	a B	30.4	a BCD	21.5	a B	1.9	a B	nd	C	2.0	ab D	25.2	a A
	Turning	18.0	a A	11.5	a B	2.1	ab B	28.3	ab B	16.0	b BC	0.9	b C	nd	C	1.7	b BC	21.5	c ABC
	Ripe	16.4	a BC	9. 6	a CD	2.0	b B	27.3	b B	18.3	ab BCD	1.2	ab C	nd	B	2.3	a BC	23.0	b A
**“Argudell”**	Green	5.3	b BC	14.6	a B	1.4	a D	32.1	a BC	21.1	a B	1.4	a B	nd	b C	3.0	a A	21.2	a CDE
	Turning	19.6	a A	10.9	b B	1.0	b CD	27.8	b B	18.1	a B	1.3	a BC	nd	b C	2.4	a AB	19.0	b C
	Ripe	20.2	a A	11.1	b C	1.4	a C	24.8	b C	19.6	a ABC	1.7	a C	0.3	a A	2.6	a BC	18.4	b E
**“Empeltre”**	Green	4.7	b BC	11.7	a CD	3.6	a A	28.9	a CD	21.8	a B	5.7	a A	0.1	a B	1.5	a E	22.1	a BC
	Turning	5.9	b C	9.5	b C	3.9	a A	26.2	a B	27.1	a A	5.6	a A	0.2	a BC	1.7	a C	20.0	a ABC
	Ripe	13.1	a CDE	8.5	c D	3.4	a A	23.1	b C	22.6	a A	5.6	a A	0.3	a AB	1.8	a DE	21.6	a ABC
**“Farga”**	Green	6.0	c B	11.2	a D	1.9	a C	26.8	a D	29.4	a A	1.6	a B	nd	b C	2.2	b CD	21.0	a CDE
	Turning	8.8	b C	11.2	a B	1.5	a BCD	28.4	a B	23.8	a A	2.0	a B	nd	b C	2.7	a A	21.7	a AB
	Ripe	19.7	a AB	9.7	a CD	1.5	a C	24.1	a C	20.8	a AB	1.2	a C	0.2	a AB	2.5	ab BC	20.3	a CD
**“Manzanilla”**	Green	5.6	b BC	17.0	a A	1.3	a D	33.9	a B	18.3	a BCD	1.3	a B	0.1	a B	2.8	a AB	19.8	a DE
	Ripe	13.3	a CD	13.9	a B	1.3	a C	28.4	b B	18.6	a ABCD	1.3	a C	0.4	a A	3.3	a A	19.5	a DE
**“Marfil”**	Green	10.2	a A	16.9	a A	2.6	a B	32.2	a BC	12.5	a D	1.8	a B	0.2	b AB	1.9	a D	21.7	a BCD
	Ripe	11.1	a DEF	14.4	b B	3.0	a A	29.5	a B	14.9	a CD	4.5	b B	0.3	a AB	1.7	a E	20.6	CD
**“Morrut”**	Green	4.3	c BC	17.7	a A	2.0	a C	32.9	a BC	13.7	a CD	1.9	a B	0.2	a A	1.9	a D	25.4	a A
	Turning	17.1	a AB	15.3	b A	1.7	a BC	28.4	a B	11.5	a C	1.6	a BC	0.4	a A	1.8	ab BC	22.2	b A
	Ripe	9.7	b EF	17.3	ab A	1.4	a C	29.7	a B	16.0	a BCD	1.8	a C	0.4	a A	1.6	b E	22.2	b AB
**“Picual”**	Green	3.3	c C	17.6	a A	0.9	b D	40.1	a A	14.7	a BCD	1.6	a B	0.1	a B	2.6	a BC	19.1	b E
	Turning	13.9	a B	15.7	b A	0.8	b D	34.5	b A	12.7	a CD	1.0	a C	0.3	a AB	2.0	b BC	19.2	b BC
	Ripe	9.6	b F	15.7	b AB	1.2	a C	34.9	b A	14.1	a D	1.4	a C	0.1	a AB	2.1	b CD	20.9	a BCD
**“Sevillenca”**	Green	5.4	b BC	13.6	a BC	2.3	a BC	31.1	a BCD	20.1	a BC	1.2	a B	0.1	a B	2.5	a BC	23.8	a AB
	Ripe	21.5	a A	11.3	b C	2.0	a B	24.3	b C	18.8	a ABCD	1.3	a C	0.3	a AB	1.8	b DE	18.7	b E

Cuticular membranes were isolated from skin samples (around 100 cm^2^) obtained from 30 to 75 olives, contingent upon fruit size. Values represent means of three technical replicates of this starting material (nd, non-detectable). Different capital letters denote significant differences among the cultivars for a given maturity stage, and different lower-case letters stand for significant differences among maturation stages for a given cultivar, at P ≤ 0.05 (Student’s t test).

* Abbreviations: FA, monocarboxylic fatty acids; α,ω-diFA, α,ω-dicarboxylic fatty acids; α,ω-diFA, mcOH, α,ω-dicarboxylic fatty acids with mid-chain-hydroxy group; ω-OH FA, ω-hydroxy fatty acids; ω-OH FA, mcOH, ω-hydroxy fatty acids with mid-chain-hydroxy group; α-OH FA, α-hydroxy fatty acids; other OH FA, other hydroxy fatty acids.

In terms of percentage over total cutin, α,ω-dicarboxylic (8.5 to 17.7%, depending on cultivar and maturity stage) and monocarboxylic (3.3 to 21.5%) fatty acids were also quantitatively important. With the exception of “Morrut” fruits, the content of dicarboxylic fatty acids decreased along maturation, while that of monocarboxylic fatty acids was significantly augmented in all the cultivars analyzed, excepting “Marfil” (**Table 5**). Within the dicarboxylic fatty acids family, 9-octadecenedioic acid was the most abundant compound identified, the highest amounts being found for “Picual” and “Morrut” olives (16.9 and 16.5%, respectively), roughly twice those determined in “Empeltre” fruit (**Supplementary Table 3**).

## Discussion

All olive samples used in this work were grown at the same orchard, under the same cultural practices. Therefore the chemical composition differences in isolated fruit cuticles among the studied cultivars are not likely to reflect different environmental conditions, and might underlie the observed features of surface topography as well as the resistance of each variety to biotic and abiotic stress factors. Water permeance of olive fruit may be modulated by different cuticle-related factors, including the presence of surface discontinuities, total wax coverage, wax-to-cutin ratio, acyclic-to-cyclic waxes ratio (which would potentially provide more efficient barriers against water loss), and ACL of acyclic wax compounds. However, no consistent relationships were found among all these variables. The toluidine test as well as cuticle yields or thickness were also apparently unrelated to water permeance values. No apparent connection was observed either between the presence of discontinuities on fruit surface as revealed by the toluidine test and the susceptibility to infestation by olive fly (*Bactrocera oleae*): among the cultivars used in this work, “Empeltre,” “Farga,” “Manzanilla,” and “Sevillenca” are characterized by severe incidence of infestation, while “Arbequina,” “Argudell,” “Marfil,” “Morrut,” and “Picual” are less susceptible to this plague (Barrios et al., 2015), which do not agree with the groupings revealed by TB staining (**Table 1**).

However, our data suggest a relationship between TB staining results and descriptors of surface roughness. Vertical roughness parameters *Stm*, *Spm*, and *Svm* can be a good indicator for uneven surfaces and for surface cracks, which will normally have higher peaks and deeper valleys. An irregular surface can also display low horizontal roughness (*S*) values, because adjacent peaks would be close to each other. Among the olive cultivars considered herein, the lowest values for horizontal roughness were observed for “Farga,” “Morrut,” and “Picual” samples, together with deeper valleys as shown by *Svm* (**Table 2**). Interestingly, when roughness parameters and TB staining data were used to characterize the samples by means of a PCA model, a good correlation was found between *S*, *Svm*, and TB test results (**Figure S5**). Eighty six percent of total variability was explained by the two first principal components (PC) alone. The plot shows that stained fruits displayed higher values for *S* and *Svm*, and the lowest for *Sa*, *Stm*, *Spm*, and *Sk*. Albeit to a lesser extent, a correlation was also found between roughness parameters and the incidence of fruit infestation by the olive fly (*B. oleae*). Higher percentage of infested fruits (**Table S1**) was associated to high *Svm* values (**Figure S5**), which might suggest that deeper irregularities on the surface may favor egg deposition.

Cuticle thickness values were higher than those reported for fruit from other olive cultivars, including “Gentile di Chieti,” “Carboncella,” “Dritta,” “Castiglionese,” “Intosso,” and “Kalamata” (Lanza and Di Serio, 2015). However, whereas that work found decreased cuticle thickness along maturation in all the cultivars assessed, a declining trend was observed in this study uniquely for “Arbequina,” “Empeltre,” “Morrut,” and “Sevillenca,” while cuticle thickness remained unchanged for the rest of cultivars considered. For tomato fruit (*S. lycopersicum* L.), cuticle thickness has been reported to increase during fruit development up to the mature green or breaker stage, and then to decrease thereafter until attaining full ripeness (Domínguez et al., 2008). Wide variation in thickness values has also been found among fruit species. Similar cuticle thickness has been reported for ripe tomato (*S. lycopersicum* L.), green and mature pepper (*Capsicum annuum* L.), and apple (*Malus pumila* L.) fruit in comparison with olive (Fernández et al., 1999), while that in mangoes (*Mangifera indica* L.) is reportedly thinner (Camacho-Vázquez et al., 2019).

A previous study on “Arbequina” (Huang et al., 2017) did not find significant differences in water permeance of fruit at different maturity stages, the observed values averaging 9.5 × 10^−5^ m s^−1^. Similar results were found in the current study for “Arbequina,” but not for all the rest of varieties: changes in water permeance throughout fruit maturation were determined for five out of the nine cultivars studied, data indicating significant increases for “Morrut” and “Picual” samples, which incidentally displayed the highest loss in water content from the green to the ripe stages (27.1 and 13.3% for “Morrut” and “Picual,” respectively) (**Table 1**). Water permeances observed in this study ranged from 6.6 to 11.9 × 10^−5^ m s^−1^, and were higher in comparison with those found for other fruit crops such as tomato (*S. lycopersicum* L.) and apple (*Malus domestica* Borkh), ranging respectively from 0.9 to 4.9 × 10^−5^ m s^−1^ (Leide et al., 2007; Leide et al., 2011) and from 4.6 to 5.3 × 10^−5^ m s^−1^ (Leide et al., 2018), but one order of magnitude lower than those observed for sweet cherry (*Prunus avium* L.) (1.4 to 3.7 × 10^−4^ m s^−1^) (Athoo et al., 2015).

With the exception of “Arbequina,” no significant changes in total cuticle yields were observed over maturation. Reports on changes in total cuticle yields (µg cm^−2^) over fruit ripening of a model species such as tomato have been shown to be cultivar-dependent (Domínguez et al., 2008; España et al., 2014), while they were found to decrease over ripening of sweet cherry (Peschel et al., 2007). Contrarily to reports for tomato (Leide et al., 2007) or orange (*Citrus sinensis* Osbeck) (Wang et al., 2016) fruit, for which increased wax coverage was observed along maturation, the opposite was found in this work for olives, with the exception of “Marfil” samples. In contrast, the proportions of the different wax fractions in olive fruit have been recently reported to be generally unrelated to sampling date, and to be largely cultivar-dependent (Vichi et al., 2016). Cutin yields were between 25.7 and 40.2%, and showed minor variations over fruit maturation. These cutin percentages over total cuticle were similar to those reported for some berries such as sea buckthorn (*Hippophaë rhamnoides* L.), cranberry (*Vaccinium oxycoccos* L.), or lingonberry (*Vaccinium vitis-idaea* L.), but much higher than those in black currant (*Ribes nigrum* L.) or bilberry (*Vaccinium myrtillus* L.) (8 and 6%, respectively) (Kallio et al., 2006). Stable cutin yields together with decreased wax coverage led to a significant decline in wax to cutin ratios (**Table 3**), in contrast with an earlier work on “Arbequina,” where no changes along fruit maturation were found (Huang et al., 2017). Both cutin yield and cutin percentage were inversely correlated to the observed olive fly egg deposition in the samples (r = −0.59 and r = −0.57 respectively), suggestive of a protective action of such compounds in the skin. This observation is in accordance with earlier suggestions that cultivar-related differences in olive fly egg deposition might be related to differential skin composition (Iannotta, 2007; Rizzo et al., 2007).

Triterpenes were the predominant cuticular wax compound type found in olive fruit as reported elsewhere (Lanza and Di Serio, 2015; Huang et al., 2017), and similarly to observations for other drupes such as sweet cherry (Peschel et al., 2007; Belge et al., 2014a) and peach (Belge et al., 2014b), as well as for blueberry (*Vaccinium* spp.) (Chu et al., 2016). Complete information on cuticle composition is available only for a handful of fruit species (reviewed in Lara et al., 2015). Whereas the triterpenoid fraction of cuticular waxes is dominated by triterpenoid alcohols in orange, Asian pear (*Pyrus sinkiangenesis* Yü and *Pyrus bretschneideri* Rehd) (Wu et al., 2017) as well as in fruit species within the *Solanaceae* family, triterpenoid acids predominate in grapes, olives, and *Rosaceae* fruit species. In fruit species in which triterpenoid acids are prevalent, the triterpenoid profile has been reported to consist uniquely of oleanolic acid (30% of total waxes) in mature grapes (*Vitis vinifera* L.) (Casado and Heredia, 1999), and of ursolic followed by oleanolic acid in peach and sweet cherry (Peschel et al., 2007; Belge et al., 2014a; Belge et al., 2014b). In olive fruit, the main triterpene compounds detected were maslinic and oleanolic acids, in agreement with previous works (Bianchi, 2003; Stiti et al., 2007; Guinda et al., 2010). An inverse relationship between triterpenoid acids and olive fly egg deposition has been reported elsewhere (Kombargi et al., 1998). Triterpenoids also have reportedly an important role in the mechanical strength of fruit cuticles (Tsubaki et al., 2013; Wu et al., 2018), and have been shown to be related to weight loss and softening rates in blueberry (Moggia et al., 2016). In this work, however, no such relationship was observed between the incidence of olive fly infestation and triterpene content (**Table 4**, **Table S1**). Contrarily, data show a positive correlation of triterpene acid levels to the percentage of affected fruit, with *r* = 0.36 and *r* = 0.49 for maslinic and oleanolic acids, respectively. Furthermore, when maturity stages were considered separately, high correlation coefficients were found for fruit at the turning stage (0.97 and 0.93 for maslinic and oleanolic acids, correspondingly). This may be relevant to understand resistance or tolerance to stress factors, as this physiological stage of the fruit coincides with environmental conditions which favor the development of pests and diseases (Vichi et al., 2016), and indeed olive fly infestation is particularly intense during the period of color change.

The profiles of fatty acids and fatty alcohols were in agreement with data reported by Huang et al. (2017), C_24_ and C_26_ being the most abundant compounds within both wax types. However, the percentage of *n*-alkanes over total waxes was very low in comparison with other fruit species: for example, *n*-alkanes accounted for 29.4% of total waxes in “Jesca” peaches at harvest (Belge et al., 2014b), whereas in the present study the highest amount detected was 4.8% in “Marfil” mature olives (**Table 4**). Accordingly, the ACL of acyclic wax compounds was lower than that found in other fruit species: ACL values were 28.8 to 29.9 in apple (Leide et al., 2018), sweet cherry (Athoo et al., 2015), and tomato (*S. lycopersicum* L.) (Leide et al., 2011).

C_18_-type cutin monomers were 2 to 3.7 times more abundant than the C_16_-type. Even so, 9/10,16-dihydroxyhexadecanoic was quantitatively the main ω-hydroxyacid with midchain hydroxyl groups identified in cutin samples, and an important compound in quantitative terms in cutin composition of all the olive cultivars considered herein. This compound has been reported to be prominent in cutin composition of mango (Camacho-Vázquez et al., 2019), a number of berries (Kallio et al., 2006; Järvinen et al., 2010), sweet cherry (Peschel et al., 2007), tomato (Leide et al., 2007), and pepper (*Capsicum* sp.) (Parsons et al., 2012). In contrast, cutin of mature persimmons (*Diospyros kaki* Thunb.) has been found to contain as much as 43.7% 9,10-epoxy-18-hydroxyoctadecanoic acid together with 17.4% 9/10,16-dihydroxyhexadecanoic acid (Tsubaki et al., 2013). In a great variety of plants, ω-hydroxy acids (either C_16_ or C_18_) dominate cutin composition (Fich et al., 2016), with agrees with results shown herein for olive fruit (**Table 5**, **Table S3**).

## Conclusions

This comparative study provided new insights in genotype-related differences in surface and cuticle features of olive fruit. Information on the chemical composition of both cuticular waxes and cutin in fruit of nine olive cultivars is reported for the first time, as well as water permeability at different maturity stages. Data on fruit micro-topography were also obtained by means of fringe projections, revealing genotype-related diversity of surface microstructure. Although water permeance of olive fruit might be controlled or fine-tuned by different cuticle-related traits, none of those considered herein appeared to suffice by itself to determine this trait, suggesting that additional properties of waxes and cutin, such as their physical structure or biomechanical properties, significantly influence the barrier functions of plant cuticles. Even so, the bulk of results reported herein should establish the basis for a better comprehension of olive crop adaptations to the surrounding environment. Further research will be paramount to elucidate the role of cuticle properties in olive resistance to plagues, rots, and adverse environmental conditions. The comprehension of these relationships would be thus very relevant for improving orchard management.

## Data Availability Statement

All datasets generated for this study are included in the article/supplementary material.

## Author Contributions

CD, JG, AR, and IL collected the samples. CD carried out the biochemical analyses. HH and MR determined cuticular transpiration. P-HL and AE were in charge of skin topography determinations. AR was responsible of the experimental orchards and the physicochemical characterization of fruit samples. TC and FG contributed to sample processing. CD and VM did the microscopy observations. CD and IL conceptualized and wrote the manuscript. All the Authors revised and approved the manuscript.

## Funding

This work was funded by grant AGL2015-64235-R from the Plan Nacional de I+D, Ministry of Education and Science, Spain. CD is the recipient of a predoctoral scholarship granted by the Universitat de Lleida. PH-L was recipient of a PhD scholarship from Zespri™ International. HH was supported by an Oversea Study Program, Guangzhou Elite Project, China.

## Conflict of Interest

The authors declare that the research was conducted in the absence of any commercial or financial relationships that could be construed as a potential conflict of interest.

## Footnotes

International Olive Council (IOC). (2018). Statistics (http://www.internationaloliveoil.org/).
