# Supplementary material for: Insights Into Olive Fruit Surface Functions: A Comparison of Cuticular Composition, Water Permeability, and Surface Topography in Nine Cultivars During Maturation
Source: Front Plant Sci. 2019 Nov 19;10:1484. doi: 10.3389/fpls.2019.01484 (PMC6878217; doi:10.3389/fpls.2019.01484)
Supplement: Supplementary file 1 [file Presentation_1.pdf]

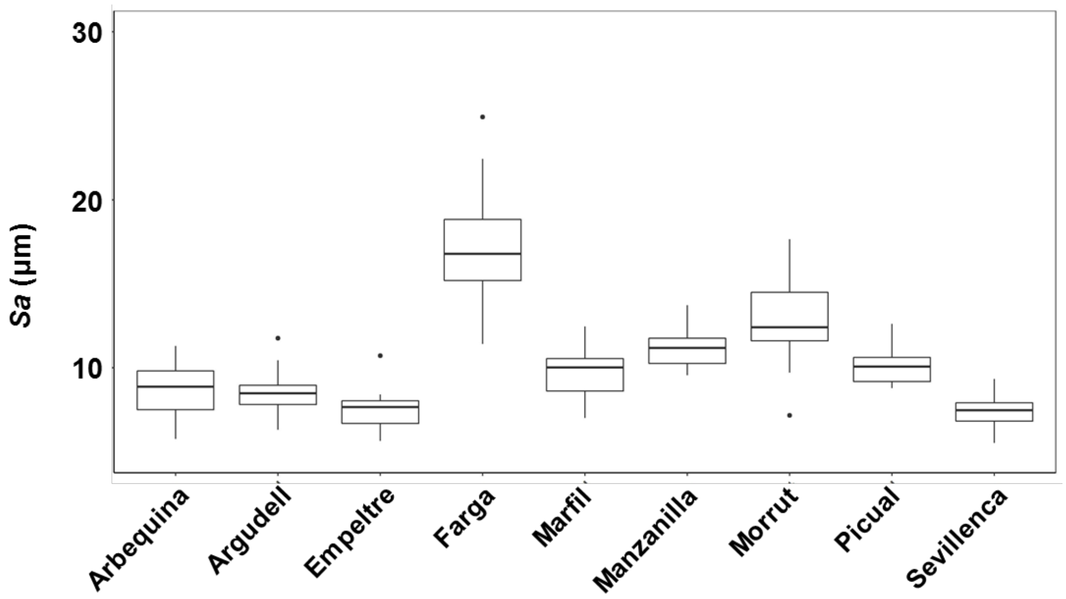

Supplementary Figure S1

Box plot of *Sa* of olive fruit surface at the green stage, as an example of differences in roughness parameters in the olive cultivars studied in this work. *Sa* is the arithmetic average height parameter, defined as the mean of the absolute deviation of roughness irregularities from the mean line.

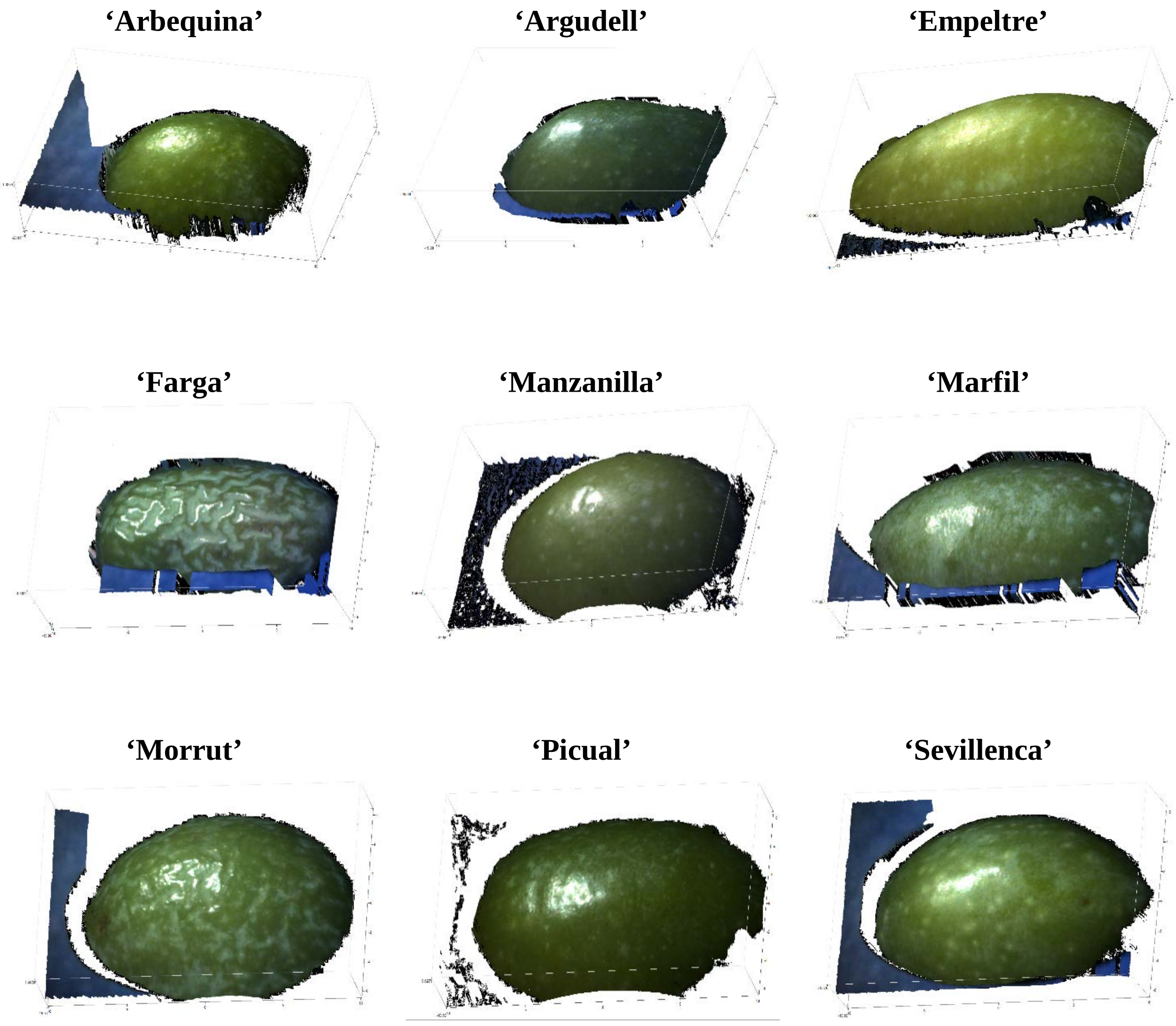

Supplementary Figure S2

3D-diagrams of raw data outputs from fringe projections of olive fruits at the green stage. Blue and black areas represent background noise due to the shape and size of the olives, which did not cover the whole assessment window of the equipment.

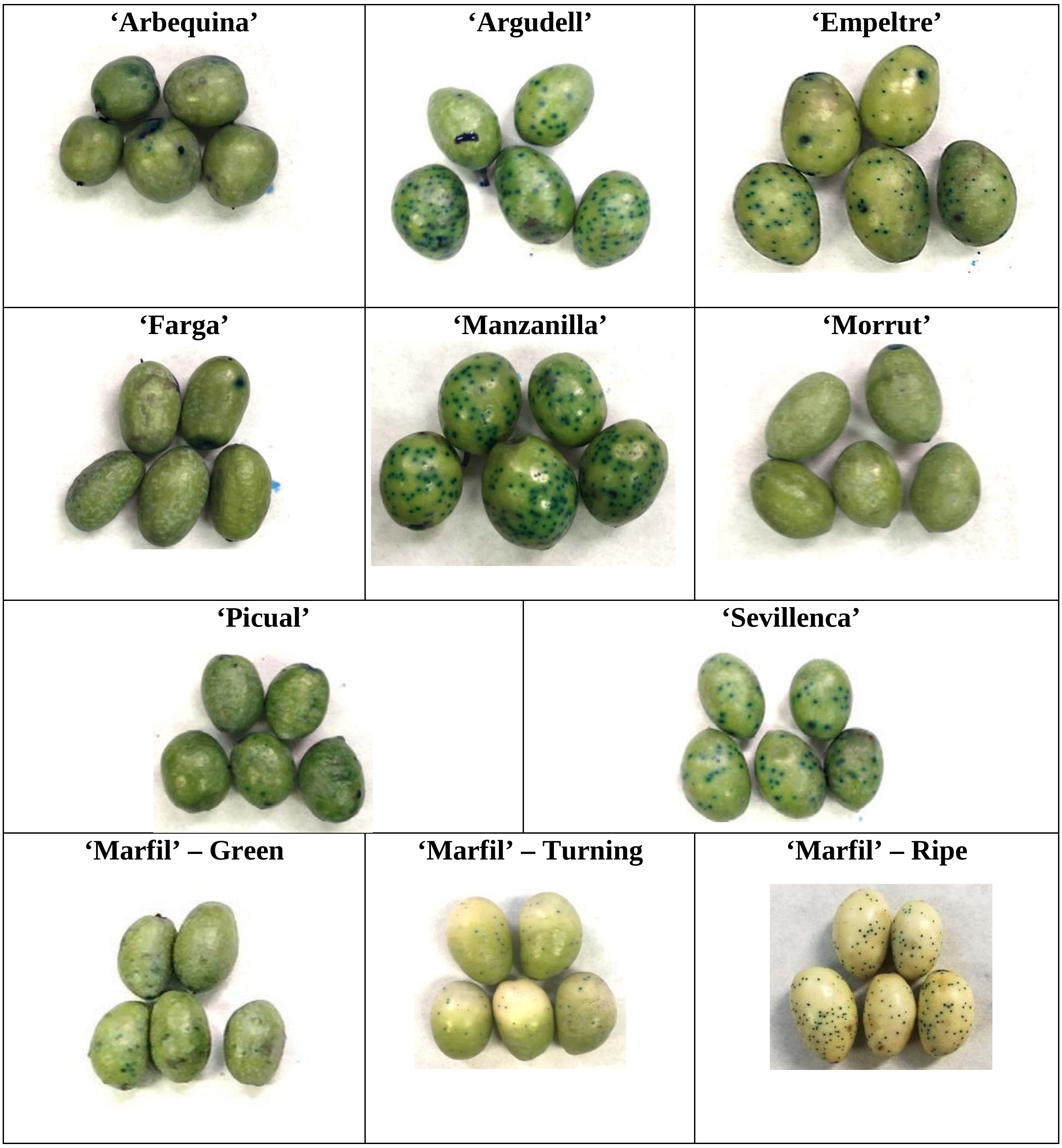

Supplementary Figure S3

Toluidine Blue (TB) staining of olive fruits at the green stage. For ‘Marfil’ fruits, TB staining is shown for samples at three different maturity stages. Fruits were stained in a 0.05% (w/v) TB solution for 2 h.

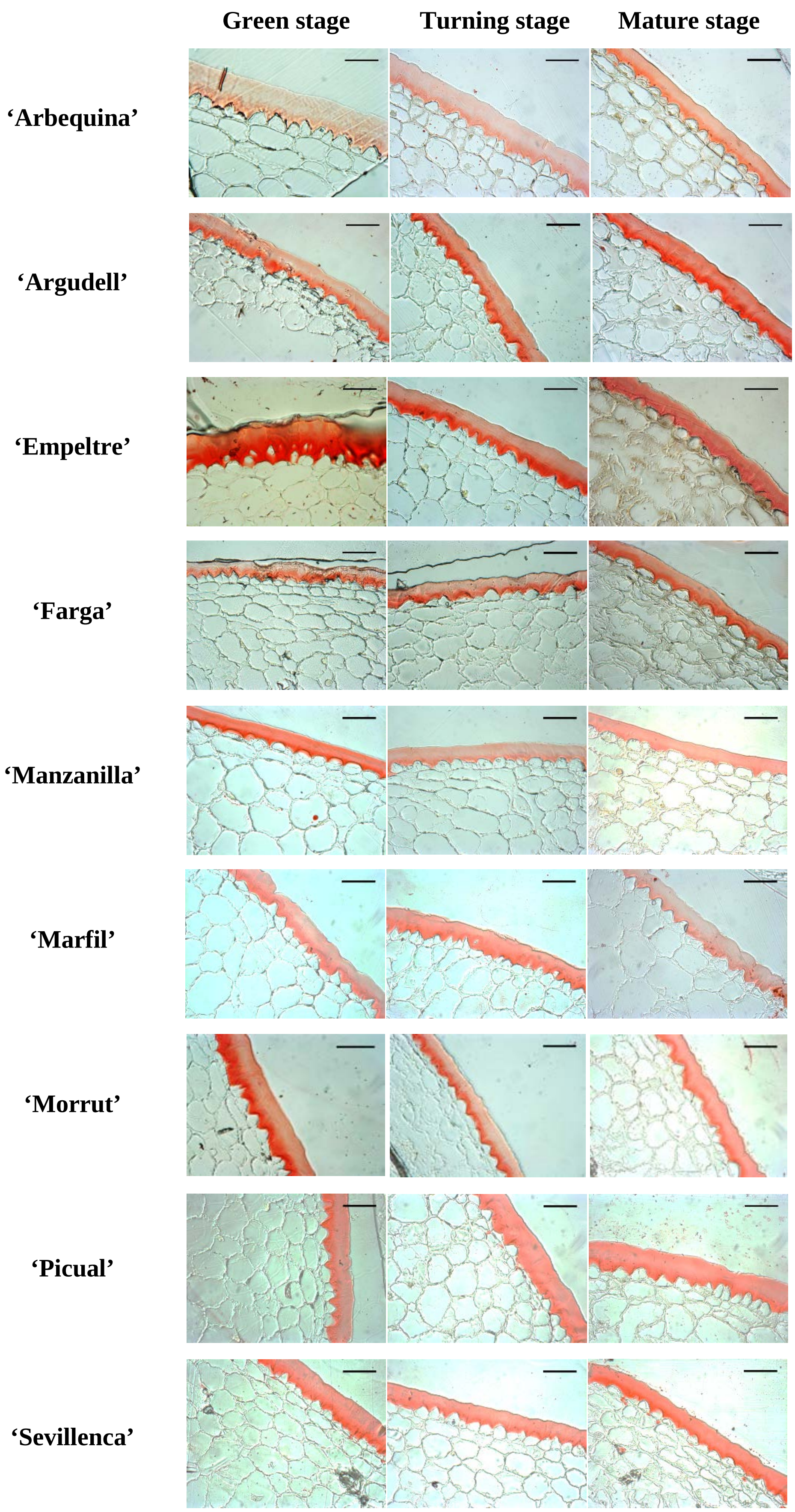

Supplementary Figure S4

Sudan IV-stained cross-sections of olive fruit pericarp at three maturity stages as observed under a bright-field microscope. The scale bars indicate 60 µm.

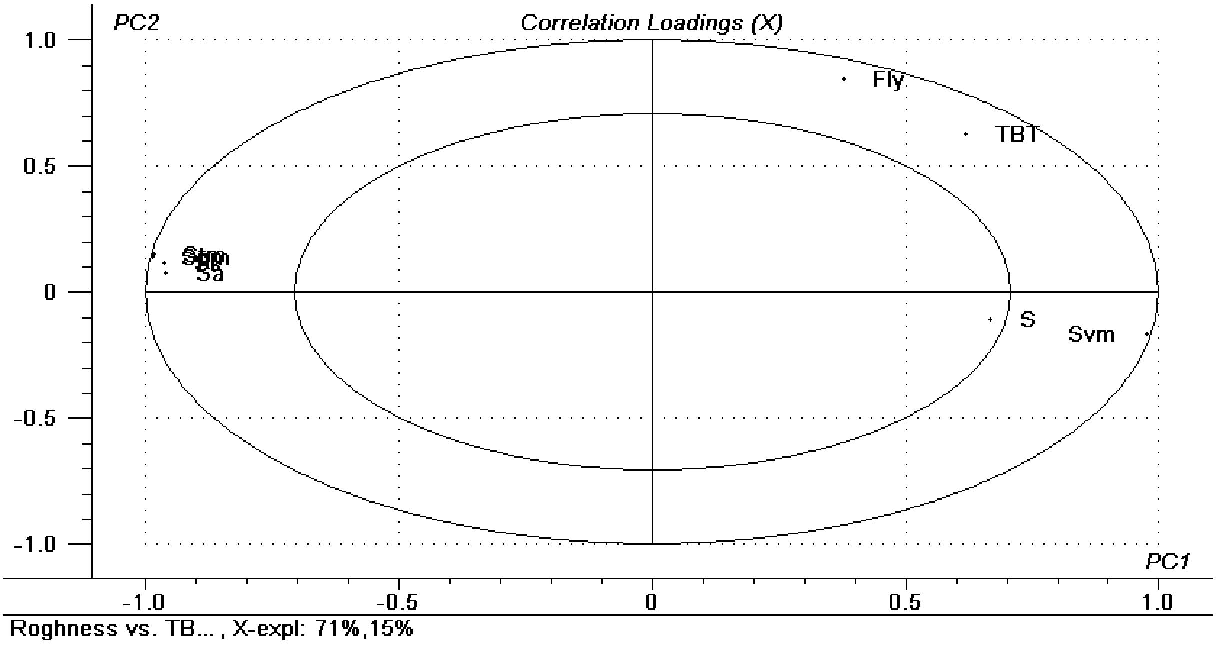

Supplementary Figure S5

Correlation loadings plot of PC1 *vs*. PC2 corresponding to a Principal Component Analysis (PCA) model for surface roughness parameters, incidence (%) of olive fly infestation (Fly) and toluidine blue test (TBT) assessed in olive fruits at the green stage. *Sa*, *Stm*, *Spm* and *Svm* are related to vertical roughness, *Sk* represents core roughness, and *S* stands for horizontal roughness as defined in Materials and Methods.
