# Supplementary material for: Insights Into Olive Fruit Surface Functions: A Comparison of Cuticular Composition, Water Permeability, and Surface Topography in Nine Cultivars During Maturation
Source: Front Plant Sci. 2019 Nov 19;10:1484. doi: 10.3389/fpls.2019.01484 (PMC6878217; doi:10.3389/fpls.2019.01484)
Supplement: Supplementary file 2 [file Table_1.pdf]

Supplementary Table 1. Incidence of infestation by *Bactrocera oleae* in olive fruits at the green, turning and ripe stages.

Cultivar	Maturity stage	Fly infestation (%)
**'Arbequina'**	Green	0
	Turning	14
	Ripe	10
**'Argudell'**	Green	12
	Turning	0
	Ripe	12
**'Empeltre'**	Green	60
	Turning	76
	Ripe	24
**'Farga'**	Green	4
	Turning	10
	Ripe	6
**'Manzanilla'**	Green	56
	Ripe	20
**'Marfil'**	Green	0
	Ripe	2
**'Morrut'**	Green	6
	Turning	0
	Ripe	4
**'Picual'**	Green	6
	Turning	6
	Ripe	8
**'Sevillenca'**	Green	24
	Ripe	0

Values represent the percentage of affected fruits within a sample of 50 olives.
