# Supplementary material for: Insights Into Olive Fruit Surface Functions: A Comparison of Cuticular Composition, Water Permeability, and Surface Topography in Nine Cultivars During Maturation
Source: Front Plant Sci. 2019 Nov 19;10:1484. doi: 10.3389/fpls.2019.01484 (PMC6878217; doi:10.3389/fpls.2019.01484)
Supplement: Supplementary file 3 [file Table_2.pdf]

Supplementary Table 2. Cuticular wax constituents (relative %) in cuticles isolated from olive fruits at the green, turning and ripe stages.

**Cultivar**	**'Arbequina'**			**'Argudell'**			**'Empeltre'**		
**Maturity stage**	**Green**	**Turning**	**Ripe**	**Green**	**Turning**	**Ripe**	**Green**	**Turning**	**Ripe**
**Fatty acids**									
C20:0	0.20 ± 0.17	1.22 ± 0.03	0.90 ± 0.14	0.23 ± 0.21	1.56 ± 0.18	1.64 ± 0.49	nd	nd	0.99 ± 0.26
C20:1 (c13)	0.17 ± 0.15	1.09 ± 0.05	0.81 ± 0.07	0.21 ± 0.19	1.31 ± 0.11	1.44 ± 0.38	nd	nd	1.06 ± 0.19
C22:0	0.64 ± 0.09	1.02 ± 0.11	1.29 ± 0.13	0.64 ± 0.06	1.32 ± 0.13	1.43 ± 0.16	0.71 ± 0.12	0.74 ± 0.09	1.65 ± 0.03
C24:0	2.69 ± 1.50	1.33 ± 0.15	2.27 ± 0.03	1.73 ± 0.32	2.26 ± 0.17	1.68 ± 0.44	1.42 ± 0.17	1.49 ± 0.36	1.90 ± 0.28
C26:0	2.63 ± 0.49	3.09 ± 0.52	3.17 ± 0.07	1.58 ± 0.20	1.63 ± 0.03	1.67 ± 0.12	2.28 ± 0.33	2.63 ± 0.46	2.65 ± 0.30
C28:0	1.04 ± 0.22	1.27 ± 0.31	1.17 ± 0.13	0.00 ± 0.00	0.35 ± 0.00	0.50 ± 0.08	0.74 ± 0.09	0.96 ± 0.17	1.06 ± 0.15
***n*-Alkanes**									
C20	0.60 ± 0.53	0.37 ± 0.02	0.31 ± 0.07	0.43 ± 0.40	0.30 ± 0.02	0.21 ± 0.05	0.27 ± 0.24	nd	0.20 ± 0.01
C21	nd	nd	nd	nd	0.23 ± 0.01	0.13 ± 0.12	nd	nd	nd
C22	0.63 ± 0.55	nd	nd	0.51 ± 0.47	nd	nd	0.45 ± 0.16	nd	nd
C25	0.62 ± 0.23	0.54 ± 0.47	0.57 ± 0.01	0.68 ± 0.31	0.73 ± 0.07	0.91 ± 0.23	0.73 ± 0.57	0.59 ± 0.34	0.90 ± 0.24
C26	0.84 ± 0.17	nd	0.47 ± 0.13	0.70 ± 0.07	0.57 ± 0.15	0.82 ± 0.15	0.70 ± 0.51	nd	0.81 ± 0.31
C27	0.43 ± 0.17	1.09 ± 0.27	0.95 ± 0.06	nd	0.43 ± 0.12	0.82 ± 0.13	nd	nd	0.81 ± 0.28
C34	0.61 ± 0.52	1.12 ± 0.66	nd	0.86 ± 0.97	0.31 ± 0.02	0.27 ± 0.07	nd	nd	nd
**Fatty alcohols**									
C22	1.43 ± 1.05	0.63 ± 0.08	0.73 ± 0.04	0.62 ± 0.07	0.49 ± 0.04	0.44 ± 0.04	1.09 ± 0.88	2.63 ± 0.63	0.52 ± 0.09
C24	1.25 ± 0.18	1.88 ± 0.59	2.31 ± 0.11	0.98 ± 0.11	1.59 ± 0.18	1.64 ± 0.19	1.00 ± 0.07	1.18 ± 1.04	1.99 ± 0.14
C26	3.83 ± 1.06	1.79 ± 0.58	1.99 ± 0.44	2.90 ± 0.66	0.69 ± 0.02	0.90 ± 0.18	2.41 ± 0.16	2.59 ± 0.61	1.04 ± 0.15
C28	1.44 ± 0.42	0.44 ± 0.40	0.70 ± 0.16	0.71 ± 0.18	nd	0.45 ± 0.16	0.63 ± 0.09	0.63 ± 0.15	nd
**Sterols**									
Squalene	0.76 ± 0.07	0.42 ± 0.38	0.41 ± 0.15	0.69 ± 0.17	0.51 ± 0.15	0.79 ± 0.11	0.63 ± 0.53	nd	0.71 ± 0.25
β-sitosterol	0.34 ± 0.31	nd	0.36 ± 0.01	0.21 ± 0.19	0.56 ± 0.16	0.89 ± 0.58	0.25 ± 0.22	0.44 ± 0.09	0.50 ± 0.12
**Triterpenes**									
Oleanolic acid	24.91 ± 3.40	23.70 ± 0.83	23.36 ± 1.13	29.59 ± 6.17	20.88 ± 0.66	21.68 ± 0.60	32.65 ± 1.25	33.94 ± 3.60	27.42 ± 0.37
Ursolic acid	nd	nd	nd	nd	nd	nd	nd	nd	nd
Maslinic acid	38.80 ± 10.83	44.55 ± 5.09	44.26 ± 2.00	40.39 ± 6.98	43.79 ± 0.77	44.55 ± 6.08	38.61 ± 0.73	39.38 ± 12.57	42.78 ± 2.18
**Unidentified**	16.15 ± 6.00	14.46 ± 1.36	13.96 ± 1.09	16.35 ± 0.99	20.48 ± 1.75	17.15 ± 2.99	15.41 ± 2.11	12.79 ± 5.65	13.00 ± 1.86

Supplementary Table 2 – Continued

**Cultivar**	**'Farga'**			**'Manzanilla'**			**'Marfil'**		
**Maturity stage**	**Green**	**Turning**	**Ripe**	**Green**	**Turning**	**Ripe**	**Green**	**Turning**	**Ripe**
**Fatty acids**									
C20:0	0.23 ± 0.21	0.74 ± 0.06	1.45 ± 0.11	0.17 ± 0.16	NA	0.79 ± 0.02	0.52 ± 0.03	NA	0.34 ± 0.04
C20:1 (c13)	0.21 ± 0.18	0.73 ± 0.05	1.57 ± 0.17	nd	NA	0.56 ± 0.00	0.80 ± 0.02	NA	0.63 ± 0.08
C22:0	0.67 ± 0.04	1.11 ± 0.13	1.67 ± 0.07	0.96 ± 0.23	NA	1.73 ± 0.12	0.49 ± 0.01	NA	1.00 ± 0.13
C24:0	2.25 ± 0.59	4.10 ± 0.48	3.04 ± 1.02	4.90 ± 1.18	NA	5.44 ± 0.57	1.00 ± 0.04	NA	2.86 ± 0.33
C26:0	2.55 ± 0.33	3.20 ± 0.53	3.41 ± 0.13	5.83 ± 1.43	NA	5.56 ± 0.74	3.52 ± 0.20	NA	4.66 ± 0.29
C28:0	0.99 ± 0.13	1.10 ± 0.18	1.58 ± 0.08	1.12 ± 0.29	NA	1.71 ± 0.14	0.98 ± 0.85	NA	2.44 ± 0.23
***n*-Alkanes**									
C20	nd	nd	nd	nd	NA	nd	nd	NA	nd
C21	nd	nd	0.15 ± 0.13	nd	NA	nd	nd	NA	nd
C22	nd	nd	nd	nd	NA	nd	nd	NA	nd
C25	0.66 ± 0.17	0.61 ± 0.10	1.09 ± 0.09	0.53 ± 0.15	NA	0.71 ± 0.09	0.51 ± 0.03	NA	0.96 ± 0.13
C26	0.29 ± 0.31	nd	0.84 ± 0.15	nd	NA	0.45 ± 0.16	nd	NA	0.56 ± 0.09
C27	0.48 ± 0.23	0.21 ± 0.19	1.38 ± 0.20	nd	NA	0.79 ± 0.22	0.28 ± 0.01	NA	3.24 ± 0.29
C34	0.53 ± 0.91	0.47 ± 0.82	nd	nd	NA	nd	0.20 ± 0.02	NA	nd
**Fatty alcohols**									
C22	0.35 ± 0.03	0.53 ± 0.28	0.48 ± 0.03	2.70 ± 0.42	NA	1.03 ± 0.08	0.41 ± 0.02	NA	0.51 ± 0.06
C24	1.35 ± 0.28	2.10 ± 0.57	2.28 ± 0.02	1.63 ± 0.65	NA	3.58 ± 0.54	0.22 ± 0.02	NA	3.19 ± 0.43
C26	2.98 ± 0.48	2.66 ± 0.69	1.37 ± 0.01	5.88 ± 1.36	NA	4.77 ± 0.71	3.44 ± 0.43	NA	2.96 ± 0.23
C28	1.33 ± 0.19	1.03 ± 0.26	0.50 ± 0.01	1.29 ± 0.30	NA	1.31 ± 0.06	1.73 ± 0.25	NA	1.41 ± 0.05
**Sterols**									
Squalene	0.32 ± 0.38	0.18 ± 0.30	0.84 ± 0.16	nd	NA	0.48 ± 0.25	0.20 ± 0.17	NA	0.55 ± 0.12
β-sitosterol	0.22 ± 0.19	0.23 ± 0.20	0.00 ± 0.00	0.28 ± 0.26	NA	0.30 ± 0.02	0.35 ± 0.08	NA	nd
**Triterpenes**									
Oleanolic acid	32.79 ± 0.96	31.70 ± 1.03	28.20 ± 0.72	24.13 ± 1.63	NA	19.14 ± 1.04	42.55 ± 1.15	NA	28.48 ± 0.76
Ursolic acid	nd	nd	1.60 ± 1.32	nd	NA	nd	nd	NA	nd
Maslinic acid	40.74 ± 3.78	38.87 ± 5.55	33.09 ± 1.51	38.98 ± 9.04	NA	39.28 ± 8.41	32.20 ± 1.58	NA	33.13 ± 3.89
**Unidentified**	11.08 ± 0.59	10.40 ± 2.41	15.46 ± 0.89	11.60 ± 1.82	NA	12.37 ± 3.72	10.59 ± 0.85	NA	13.09 ± 1.58

Supplementary Table 2 – Continued

**Cultivar**	**'Morrut'**			**'Picual'**			**'Sevillenca'**		
**Maturity stage**	**Green**	**Turning**	**Ripe**	**Green**	**Turning**	**Ripe**	**Green**	**Turning**	**Ripe**
**Fatty acids**									
C20:0	0.20 ± 0.18	2.19 ± 0.16	0.88 ± 0.15	nd	1.20 ± 0.14	0.60 ± 0.04	nd	NA	1.75 ± 0.24
C20:1 (c13)	nd	1.37 ± 0.08	0.87 ± 0.08	nd	0.76 ± 0.09	0.41 ± 0.02	nd	NA	1.50 ± 0.23
C22:0	1.05 ± 0.18	2.03 ± 0.24	1.44 ± 0.17	0.58 ± 0.10	1.00 ± 0.19	0.84 ± 0.15	1.22 ± 0.13	NA	1.38 ± 0.03
C24:0	4.58 ± 0.88	4.65 ± 0.21	3.94 ± 0.93	1.34 ± 0.04	2.26 ± 0.77	1.29 ± 0.05	5.48 ± 0.83	NA	3.60 ± 0.52
C26:0	0.33 ± 0.30	4.18 ± 0.38	4.36 ± 0.63	0.92 ± 0.10	1.45 ± 0.27	1.10 ± 0.09	7.16 ± 1.24	NA	4.90 ± 0.54
C28:0	nd	0.79 ± 0.07	1.22 ± 0.12	nd	nd	0.34 ± 0.09	1.81 ± 0.30	NA	1.92 ± 0.46
***n*-Alkanes**									
C20	nd	nd	nd	nd	nd	nd	nd	NA	nd
C21	nd	0.26 ± 0.03	nd	nd	0.13 ± 0.11	0.16 ± 0.14	nd	NA	0.28 ± 0.03
C22	nd	nd	nd	nd	nd	nd	nd	NA	nd
C25	0.46 ± 0.12	0.62 ± 0.05	0.63 ± 0.05	0.16 ± 0.14	0.75 ± 0.09	0.48 ± 0.07	0.29 ± 0.25	NA	0.72 ± 0.10
C26	nd	0.37 ± 0.15	0.29 ± 0.04	nd	0.63 ± 0.16	0.50 ± 0.17	0.24 ± 0.21	NA	0.48 ± 0.25
C27	nd	0.69 ± 0.19	1.27 ± 0.12	nd	0.70 ± 0.16	0.67 ± 0.18	nd	NA	0.82 ± 0.43
C34	nd	nd	nd	nd	nd	nd	1.17 ± 0.06	NA	nd
**Fatty alcohols**									
C22	0.71 ± 0.09	0.28 ± 0.14	0.49 ± 0.03	1.07 ± 0.53	0.67 ± 0.08	1.04 ± 0.68	3.15 ± 0.27	NA	0.33 ± 0.04
C24	1.20 ± 0.16	1.77 ± 0.15	1.97 ± 0.13	0.80 ± 0.09	1.07 ± 0.23	1.11 ± 0.19	1.26 ± 0.25	NA	1.37 ± 0.17
C26	nd	1.22 ± 0.10	1.98 ± 0.34	2.45 ± 0.15	1.59 ± 0.29	1.28 ± 0.14	3.74 ± 0.40	NA	0.76 ± 0.27
C28	0.56 ± 0.12	nd	0.56 ± 0.08	0.19 ± 0.16	nd	0.21 ± 0.02	1.69 ± 0.19	NA	0.48 ± 0.27
**Sterols**									
Squalene	nd	0.39 ± 0.16	nd	nd	0.61 ± 0.12	0.66 ± 0.16	0.60 ± 0.04	NA	0.62 ± 0.36
β-sitosterol	0.23 0.20	1.10 ± 0.93	0.36 ± 0.03	nd	0.85 ± 0.12	0.50 ± 0.04	0.29 ± 0.25	NA	0.84 ± 0.57
**Triterpenes**									
Oleanolic acid	27.07 ± 2.38	21.00 ± 1.81	20.60 ± 0.89	29.06 ± 1.84	29.63 ± 1.79	24.74 ± 0.81	33.78 ± 1.10	NA	19.32 ± 1.02
Ursolic acid	nd	nd	nd	nd	nd	0.44 ± 0.47	1.22 ± 0.43	NA	nd
Maslinic acid	43.63 ± 7.18	43.13 ± 2.71	46.64 ± 1.79	51.71 ± 3.63	42.50 ± 3.53	36.73 ± 9.08	26.47 ± 4.20	NA	38.79 ± 6.01
**Unidentified**	19.97 ± 3.11	13.95 ± 0.67	12.51 ± 0.81	11.74 ± 1.77	14.20 ± 0.56	26.89 ± 8.74	10.43 ± 0.17	NA	20.13 ± 3.45

Cuticular membranes were isolated from skin samples (around 100 cm^2) obtained from 30 to 75 olives, contingent upon fruit size. Values represent means of three technical replicates of this starting material ± standard deviation (nd, non-detectable; NA, value not available).
