# Supplementary material for: Insights Into Olive Fruit Surface Functions: A Comparison of Cuticular Composition, Water Permeability, and Surface Topography in Nine Cultivars During Maturation
Source: Front Plant Sci. 2019 Nov 19;10:1484. doi: 10.3389/fpls.2019.01484 (PMC6878217; doi:10.3389/fpls.2019.01484)
Supplement: Supplementary file 4 [file Table_3.pdf]

Supplementary Table 3. Cutin constituents (relative %) in cuticles isolated from olive fruits at the green, turning and ripe stages.

**Cultivar**	**'Arbequina'**			**'Argudell'**			**'Empeltre'**		
**Maturity stage**	**Green**	**Turning**	**Ripe**	**Green**	**Turning**	**Ripe**	**Green**	**Turning**	**Ripe**
**Monocarboxylic fatty acids**									
C16:0	0.92 ± 0.08	2.78 ± 0.11	2.46 ± 0.32	0.90 ± 0.04	3.17 ± 0.19	3.21 ± 0.70	0.59 ± 0.03	0.94 ± 0.56	1.73 ± 0.22
C16:1 (c9)	0.17 ± 0.03	0.33 ± 0.02	0.32 ± 0.04	0.15 ± 0.00	0.26 ± 0.01	0.35 ± 0.06	nd	nd	nd
C18:0	0.20 ± 0.03	0.39 ± 0.04	0.31 ± 0.04	0.20 ± 0.05	0.47 ± 0.05	0.52 ± 0.23	0.16 ± 0.03	0.42 ± 0.48	0.34 ± 0.13
C18:1 (c9)	1.84 ± 0.28	9.87 ± 0.74	8.46 ± 1.05	2.09 ± 0.12	9.31 ± 0.60	10.82 ± 1.99	1.55 ± 0.15	2.27 ± 1.27	6.86 ± 0.80
C18:1 (t9)	0.12 ± 0.02	0.48 ± 0.02	0.48 ± 0.05	0.12 ± 0.00	0.46 ± 0.03	0.64 ± 0.18	nd	0.25 ± 0.26	0.36 ± 0.09
C18:2 (c9,c12)	0.65 ± 0.11	2.23 ± 0.16	1.91 ± 0.24	0.70 ± 0.05	2.69 ± 0.23	2.94 ± 0.55	0.58 ± 0.04	0.76 ± 0.27	1.76 ± 0.06
C20:0	nd	nd	nd	nd	0.32 ± 0.05	0.23 ± 0.10	nd	nd	nd
C20:1 (c13)	nd	nd	nd	0.18 ± 0.00	nd	nd	nd	nd	nd
C22:0	0.58 ± 0.57	1.43 ± 0.24	1.88 ± 0.07	0.68 ± 0.38	1.68 ± 0.53	1.20 ± 0.27	1.03 ± 0.02	0.61 ± 0.13	1.44 ± 0.37
C24:0	0.53 ± 0.08	0.30 ± 0.03	0.30 ± 0.04	0.30 ± 0.06	0.66 ± 0.48	0.24 ± 0.03	0.35 ± 0.07	0.33 ± 0.09	0.29 ± 0.01
C26:0	nd	0.15 ± 0.13	0.22 ± 0.02	nd	0.61 ± 0.75	nd	0.38 ± 0.06	0.28 ± 0.02	0.36 ± 0.04
C28:0	nd	nd	nd	nd	nd	nd	nd	nd	nd
**α,ω-Dicarboxylic fatty acids**									
C16:0	0.81 ± 0.00	0.69 ± 0.06	0.68 ± 0.03	0.96 ± 0.11	0.78 ± 0.01	0.73 ± 0.06	0.79 ± 0.07	0.82 ± 0.11	0.61 ± 0.04
C18:1 (c9)	10.65 ± 1.40	10.76 ± 0.89	8.87 ± 1.45	13.61 ± 1.93	10.08 ± 0.15	10.38 ± 1.24	10.87 ± 0.36	8.66 ± 0.41	7.92 ± 0.20
**α,ω-Dicarboxylic fatty acids with mid-chain-hydroxy group**									
C18:0 (9,10-diOH)	2.63 ± 0.09	2.12 ± 0.31	1.97 ± 0.39	1.36 ± 0.05	0.95 ± 0.13	1.42 ± 0.25	3.58 ± 0.23	3.90 ± 0.95	3.40 ± 0.16
**ω-Hydroxy fatty acids**									
C16:0	7.96 ± 0.32	6.97 ± 0.54	7.02 ± 0.35	9.95 ± 0.41	8.79 ± 0.78	7.03 ± 0.65	6.12 ± 0.42	6.11 ± 0.60	4.98 ± 0.09
C18:0	4.00 ± 0.23	3.97 ± 0.31	4.02 ± 0.40	2.50 ± 0.38	3.55 ± 0.21	2.91 ± 0.33	5.07 ± 0.09	4.52 ± 0.33	4.23 ± 0.13
C18:1 (c9)	15.87 ± 2.04	15.58 ± 1.00	13.67 ± 1.79	17.79 ± 1.80	13.47 ± 0.48	13.14 ± 0.99	15.58 ± 0.98	13.40 ± 1.02	11.99 ± 0.22
C18:2 (c9,c12)	2.26 ± 0.30	1.82 ± 0.13	2.21 ± 0.49	1.85 ± 0.32	2.01 ± 0.17	1.37 ± 0.25	1.89 ± 0.23	1.85 ± 0.16	1.73 ± 0.14
C20:0	0.29 ± 0.08	nd	0.42 ± 0.04	nd	nd	0.33 ± 0.05	0.24 ± 0.08	0.32 ± 0.14	0.19 ± 0.04
**ω-Hydroxy fatty acids with mid-chain-hydroxy group**									
C16:0 (9/10,16-diOH)	18.91 ± 3.85	13.29 ± 2.19	15.86 ± 2.65	19.57 ± 2.63	17.26 ± 1.85	19.15 ± 5.02	16.73 ± 1.74	23.14 ± 5.72	19.03 ± 1.47
C18:0 (9,10,18-triOH)	2.57 ± 0.78	2.70 ± 0.12	2.40 ± 0.25	1.49 ± 0.10	0.85 ± 0.48	0.41 ± 0.08	5.08 ± 0.31	4.00 ± 0.46	3.58 ± 0.19
**α-Hydroxy fatty acids**									
C22:0 (2-OH)	1.87 ± 0.49	0.93 ± 0.29	1.24 ± 0.08	1.35 ± 0.07	1.32 ± 0.35	1.41 ± 0.52	5.73 ± 0.37	5.59 ± 0.93	5.35 ± 0.52
C24:0 (2-OH)	nd	nd	nd	nd	nd	0.25 ± 0.19	nd	nd	0.21 ± 0.16
**Other hydroxy fatty acids**									
C18:1 (c9, 12-OH)	nd	nd	nd	nd	nd	0.34 ± 0.38	nd	nd	0.26 ± 0.25
C18:1 (c9, 17-OH)	nd	nd	nd	nd	nd	nd	0.12 ± 0.10	0.18 ± 0.11	nd

**Fatty alcohols**									
C24	1.01 ± 0.30	0.88 ± 0.10	1.29 ± 0.13	1.09 ± 0.13	1.00 ± 0.24	1.31 ± 0.36	1.03 ± 0.11	1.22 ± 0.12	1.34 ± 0.25
C26	0.74 ± 0.09	0.65 ± 0.07	0.81 ± 0.14	1.66 ± 0.15	1.11 ± 0.65	1.12 ± 0.18	0.44 ± 0.06	0.43 ± 0.04	0.42 ± 0.05
C28	0.20 ± 0.03	0.19 ± 0.02	0.22 ± 0.02	0.26 ± 0.02	0.25 ± 0.02	0.18 ± 0.02	nd	nd	nd
**Unidentified**	25.23 ± 0.78	21.50 ± 0.55	22.97 ± 0.76	21.23 ± 1.15	18.96 ± 1.18	18.39 ± 0.81	22.08 ± 0.56	20.00 ± 3.08	21.61 ± 0.40

Supplementary Table 3 – Continued

Cultivar	'Farga'			'Manzanilla'			'Marfil'		
**Maturity stage**	**Green**	**Turning**	**Ripe**	**Green**	**Turning**	**Ripe**	**Green**	**Turning**	**Ripe**
**Monocarboxylic fatty acids**									
C16:0	0.66 ± 0.09	1.04 ± 0.11	1.98 ± 0.07	0.87 ± 0.26	NA	1.86 ± 0.28	2.00 ± 1.43	NA	1.34 ± 0.49
C16:1 (c9)	nd	nd	nd	nd	NA	nd	nd	NA	nd
C18:0	0.19 ± 0.02	0.23 ± 0.02	0.46 ± 0.03	0.31 ± 0.22	NA	0.55 ± 0.09	0.94 ± 0.96	NA	0.24 ± 0.13
C18:1 (c9)	2.51 ± 0.36	4.44 ± 0.46	12.41 ± 0.41	2.06 ± 0.70	NA	6.61 ± 1.17	3.84 ± 1.05	NA	6.03 ± 1.70
C18:1 (t9)	nd	0.17 ± 0.02	0.38 ± 0.01	nd	NA	0.36 ± 0.07	0.18 ± 0.05	NA	0.24 ± 0.05
C18:2 (c9,c12)	0.50 ± 0.07	0.89 ± 0.09	2.34 ± 0.19	0.60 ± 0.16	NA	1.33 ± 0.18	0.62 ± 0.22	NA	1.70 ± 0.42
C20:0	nd	nd	0.20 ± 0.03	nd	NA	0.27 ± 0.06	nd	NA	nd
C20:1 (c13)	nd	nd	nd	0.20 ± 0.01	NA	0.17 ± 0.01	0.16 ± 0.01	NA	nd
C22:0	1.52 ± 0.20	1.40 ± 0.62	1.43 ± 0.24	1.03 ± 0.08	NA	1.45 ± 0.90	1.77 ± 0.86	NA	1.00 ± 0.39
C24:0	0.38 ± 0.06	0.36 ± 0.06	0.26 ± 0.03	0.25 ± 0.04	NA	0.23 ± 0.05	0.42 ± 0.04	NA	0.23 ± 0.01
C26:0	0.25 ± 0.03	0.26 ± 0.01	0.26 ± 0.01	0.23 ± 0.02	NA	0.27 ± 0.02	0.30 ± 0.06	NA	0.31 ± 0.01
C28:0	nd	nd	nd	nd	NA	0.15 ± 0.02	nd	NA	nd
**α,ω-Dicarboxylic fatty acids**									
C16:0	0.75 ± 0.06	0.78 ± 0.05	0.60 ± 0.01	1.67 ± 0.22	NA	1.30 ± 0.10	1.88 ± 0.21	NA	1.34 ± 0.05
C18:1 (c9)	10.43 ± 1.46	10.45 ± 0.43	9.10 ± 0.36	15.29 ± 0.54	NA	12.59 ± 1.93	15.05 ± 0.61	NA	13.10 ± 0.84
**α,ω-Dicarboxylic fatty acids with mid-chain-hydroxy group**									
C18:0 (9,10-diOH)	1.93 ± 0.70	1.45 ± 0.09	1.46 ± 0.10	1.30 ± 0.24	NA	1.33 ± 0.12	2.61 ± 0.27	NA	2.98 ± 0.30
**ω-Hydroxy fatty acids**									
C16:0	7.19 ± 0.96	7.35 ± 0.26	5.91 ± 0.10	13.12 ± 0.49	NA	11.09 ± 0.13	7.86 ± 0.39	NA	6.01 ± 0.37
C18:0	3.99 ± 2.46	4.62 ± 0.22	4.00 ± 0.27	2.61 ± 1.34	NA	2.59 ± 0.67	2.49 ± 1.15	NA	4.51 ± 0.16
C18:1 (c9)	12.41 ± 2.20	13.32 ± 0.59	11.53 ± 0.13	16.04 ± 0.14	NA	12.67 ± 1.21	19.80 ± 0.37	NA	17.20 ± 0.98
C18:2 (c9,c12)	3.15 ± 0.28	3.14 ± 0.20	2.67 ± 0.26	1.79 ± 0.20	NA	1.77 ± 0.21	1.77 ± 0.11	NA	1.56 ± 0.06
C20:0	nd	nd	nd	0.36 ± 0.08	NA	0.31 ± 0.05	0.27 ± 0.02	NA	0.23 ± 0.07
**ω-Hydroxy fatty acids with mid-chain-hydroxy group**									
C16:0 (9/10,16-diOH	27.42 ± 9.65	22.03 ± 2.43	19.00 ± 0.96	16.85 ± 2.33	NA	16.90 ± 3.38	9.36 ± 1.88	NA	11.66 ± 1.52
C18:0 (9,10,18-triOH	1.98 ± 0.25	1.75 ± 0.15	1.78 ± 0.01	1.40 ± 0.20	NA	1.71 ± 0.12	3.09 ± 0.20	NA	3.26 ± 0.08
**α-Hydroxy fatty acids**									
C22:0 (2-OH)	1.60 ± 0.35	1.95 ± 0.50	1.23 ± 0.46	1.30 ± 0.50	NA	1.10 ± 0.50	1.75 ± 0.91	NA	4.46 ± 0.33
C24:0 (2-OH)	nd	nd	nd	nd	NA	0.23 ± 0.05	nd	NA	nd
**Other hydroxy fatty acids**									
C18:1 (c9, 12-OH)	nd	nd	0.17 ± 0.01	nd	NA	0.22 ± 0.21	nd	NA	0.14 ± 0.02
C18:1 (c9, 17-OH)	nd	nd	nd	0.12 ± 0.01	NA	0.17 ± 0.04	0.16 ± 0.01	NA	0.17 ± 0.01

**Fatty alcohols**									
C24	0.99 ± 0.13	1.49 ± 0.25	1.36 ± 0.10	0.96 ± 0.12	NA	1.39 ± 0.31	0.54 ± 0.19	NA	0.84 ± 0.17
C26	0.84 ± 0.16	0.91 ± 0.09	0.87 ± 0.14	1.61 ± 0.03	NA	1.57 ± 0.08	0.99 ± 0.03	NA	0.62 ± 0.02
C28	0.33 ± 0.06	0.31 ± 0.03	0.30 ± 0.05	0.26 ± 0.02	NA	0.30 ± 0.02	0.40 ± 0.02	NA	0.21 ± 0.02
**Unidentified**	20.97 ± 1.93	21.67 ± 1.02	20.30 ± 0.97	19.76 ± 0.84	NA	19.49 ± 1.06	21.73 ± 2.31	NA	20.63 ± 0.32

Supplementary Table 3 – Continued

**Cultivar**	**'Morrut'**			**'Picual'**			**'Sevillenca'**		
**Maturity stage**	**Green**	**Turning**	**Ripe**	**Green**	**Turning**	**Ripe**	**Green**	**Turning**	**Ripe**
**Monocarboxylic fatty acids**									
C16:0	0.43 ± 0.01	1.73 ± 0.06	1.10 ± 0.16	0.38 ± 0.03	1.75 ± 0.35	1.16 ± 0.10	0.80 ± 0.03	NA	2.77 ± 0.11
C16:1 (c9)	nd	nd	nd	nd	nd	nd	nd	NA	nd
C18:0	0.18 ± 0.05	0.64 ± 0.04	0.29 ± 0.08	0.14 ± 0.02	0.49 ± 0.07	0.36 ± 0.03	0.18 ± 0.01	NA	0.56 ± 0.10
C18:1 (c9)	1.32 ± 0.03	10.09 ± 0.75	5.09 ± 0.70	1.13 ± 0.16	9.20 ± 1.97	6.14 ± 0.54	1.67 ± 0.05	NA	12.33 ± 0.38
C18:1 (t9)	nd	0.19 ± 0.04	0.15 ± 0.05	nd	0.26 ± 0.04	0.18 ± 0.02	nd	NA	0.39 ± 0.06
C18:2 (c9,c12)	0.69 ± 0.04	2.65 ± 0.22	1.69 ± 0.25	0.29 ± 0.02	0.92 ± 0.20	0.60 ± 0.04	0.72 ± 0.01	NA	3.37 ± 0.07
C20:0	nd	0.21 ± 0.04	0.17 ± 0.04	nd	0.12 ± 0.01	nd	nd	NA	nd
C20:1 (c13)	0.24 ± 0.02	0.18 ± 0.00	0.16 ± 0.02	0.23 ± 0.01	0.19 ± 0.01	0.19 ± 0.01	nd	NA	nd
C22:0	0.92 ± 0.56	0.92 ± 0.24	0.46 ± 0.22	0.88 ± 0.19	0.78 ± 0.31	0.77 ± 0.09	1.39 ± 0.17	NA	1.51 ± 0.28
C24:0	0.28 ± 0.02	0.24 ± 0.01	0.24 ± 0.04	0.21 ± 0.02	0.18 ± 0.01	0.21 ± 0.02	0.34 ± 0.02	NA	0.25 ± 0.00
C26:0	0.21 ± 0.02	0.23 ± 0.02	0.21 ± 0.02	nd	nd	nd	0.30 ± 0.03	NA	0.30 ± 0.03
C28:0	nd	nd	0.17 ± 0.03	nd	nd	nd	nd	NA	nd
**α,ω-Dicarboxylic fatty acids**									
C16:0	0.86 ± 0.07	0.72 ± 0.03	1.06 ± 0.04	0.74 ± 0.02	0.67 ± 0.07	0.63 ± 0.02	1.16 ± 0.12	NA	0.87 ± 0.03
C18:1 (c9)	16.88 ± 1.08	14.61 ± 0.75	16.20 ± 1.29	16.88 ± 0.53	15.04 ± 0.17	15.05 ± 0.34	12.39 ± 0.72	NA	10.39 ± 0.20
**α,ω-Dicarboxylic fatty acids with mid-chain-hydroxy group**									
C18:0 (9,10-diOH)	1.95 ± 0.40	1.68 ± 0.13	1.38 ± 0.48	0.90 ± 0.07	0.77 ± 0.05	1.19 ± 0.15	2.33 ± 0.23	NA	2.02 ± 0.13
**ω-Hydroxy fatty acids**									
C16:0	5.87 ± 0.84	4.88 ± 0.13	6.65 ± 0.66	12.01 ± 0.67	9.96 ± 0.86	9.12 ± 0.38	9.20 ± 0.59	NA	6.79 ± 0.68
C18:0	2.01 ± 0.96	2.47 ± 0.16	2.23 ± 0.17	2.50 ± 0.15	1.92 ± 0.30	2.26 ± 0.17	3.34 ± 0.13	NA	2.72 ± 0.19
C18:1 (c9)	23.54 ± 2.50	19.78 ± 0.59	19.55 ± 2.09	24.12 ± 0.54	21.48 ± 1.14	22.54 ± 1.11	16.46 ± 0.53	NA	13.46 ± 0.59
C18:2 (c9,c12)	1.28 ± 0.13	0.97 ± 0.01	1.06 ± 0.03	1.20 ± 0.12	0.93 ± 0.17	0.99 ± 0.13	1.82 ± 0.07	NA	1.38 ± 0.14
C20:0	0.22 ± 0.02	0.26 ± 0.01	0.24 ± 0.04	0.25 ± 0.05	0.19 ± 0.01	nd	0.23 ± 0.04	NA	nd
**ω-Hydroxy fatty acids with mid-chain-hydroxy group**									
C16:0 (9/10,16-diOH	11.45 ± 2.36	9.39 ± 1.09	14.31 ± 5.18	13.60 ± 1.65	11.83 ± 0.39	13.68 ± 2.35	17.83 ± 1.00	NA	16.99 ± 0.68
C18:0 (9,10,18-triOH	2.23 ± 0.26	2.13 ± 0.29	1.64 ± 0.39	1.14 ± 0.04	0.85 ± 0.09	0.44 ± 0.03	2.29 ± 0.31	NA	1.81 ± 0.12
**α-Hydroxy fatty acids**									
C22:0 (2-OH)	1.87 ± 0.43	1.52 ± 0.30	1.63 ± 0.06	1.60 ± 0.62	0.83 ± 0.31	1.41 ± 0.34	1.20 ± 0.31	1.87 NA	1.02 ± 0.13
C24:0 (2-OH)	nd	0.12 ± 0.10	0.16 ± 0.06	nd	0.14 ± 0.07	nd	nd	nd NA	0.24 ± 0.13
**Other hydroxy fatty acids**									
C18:1 (c9, 12-OH)	nd	0.20 ± 0.16	0.18 ± 0.13	nd	0.17 ± 0.14	nd	nd	NA	0.30 ± 0.17
C18:1 (c9, 17-OH)	0.22 ± 0.01	0.19 ± 0.02	0.19 ± 0.03	0.13 ± 0.00	0.12 ± 0.01	0.11 ± 0.01	0.12 ± 0.01	NA	nd

**Fatty alcohols**									
C24	0.99 ± 0.14	1.22 ± 0.11	1.05 ± 0.05	1.02 ± 0.16	0.80 ± 0.05	1.01 ± 0.17	1.03 ± 0.19	NA	0.86 ± 0.11
C26	0.78 ± 0.12	0.55 ± 0.01	0.58 ± 0.12	1.39 ± 0.11	1.11 ± 0.03	1.11 ± 0.09	1.06 ± 0.02	NA	0.71 ± 0.09
C28	0.16 ± 0.02	nd	nd	0.15 ± 0.01	0.13 ± 0.01	nd	0.37 ± 0.02	NA	0.23 ± 0.02
**Unidentified**	25.42 ± 1.04	22.23 ± 0.43	22.17 ± 1.26	19.10 ± 0.34	19.17 ± 1.01	20.86 ± 0.27	23.79 ± 0.45	NA	18.74 ± 0.90

Cuticular membranes were isolated from skin samples (around 100 cm^2) obtained from 30 to 75 olives, contingent upon fruit size. Values represent means of three technical replicates of this starting material ± standard deviation (nd, non-detectable; NA, value not available).
